# *In Silico* and *In Vitro* Characterization of Fecal Spore-Forming Bacteria with Probiotic Potential

**DOI:** 10.3390/ijms27167364

**Published:** 2026-08-18

**Authors:** Rafaella Sinnott Dias, Ana Vitória Costa, Mariana Silveira Mello Silva, Anella Saggese, Ylenia De Luca, Loredana Baccigalupi, Frederico Schmitt Kremer, Fábio Pereira Leivas Leite, Luciana Farias da Costa de Avila, Ezio Ricca

**Affiliations:** 1 Núcleo Biotecnologia, Centro de Desenvolvimento Tecnológico, Universidade Federal de Pelotas, Pelotas 96050-500, RS, Brazil; rafaellasinnott@gmail.com (R.S.D.); anavitoriacost4@gmail.com (A.V.C.); fred.s.kremer@gmail.com (F.S.K.); fleivasleite@gmail.com (F.P.L.L.); 2 Laboratório de Parasitologia, Faculdade de Medicina, Universidade Federal do Rio Grande, Porto Alegre 90040-060, RS, Brazil; marianamello447@gmail.com (M.S.M.S.); lucostaavila@hotmail.com (L.F.d.C.d.A.); 3 Department of Biology, Federico II University of Naples, 80126 Naples, Italy; anella.saggese@unina.it (A.S.); ylenia.deluca@unina.it (Y.D.L.); 4 Department of Molecular Medicine and Medical Biotechnology, Federico II University of Naples, 80131 Naples, Italy; lorbacci@unina.it

**Keywords:** *Bacillus spizizenii*, *Priestia megaterium*, antimicrobial activity, antibiofilm activity, genome mining, next-generation probiotics

## Abstract

The search for next-generation probiotics derived from the human gut microbiota has intensified due to their potential beneficial effects in modulating immunity and preventing diseases. Spore-forming bacteria are attractive probiotic candidates due to their functional traits and unique resilience, which offer high stability to commercial formulations. In this study, spore-forming bacteria isolated from human fecal samples were screened. Two strains, *Bacillus spizizenii* A1 and *Priestia megaterium* F6, were identified as non-hemolytic and not resistant to a panel of antibiotics relevant for spore formers. Both strains were characterized by using *in silico* and *in vitro* approaches, demonstrating relevant probiotic potential. The comparative analysis indicated *B. spizizenii* A1 as the most promising probiotic candidate due to the resistance of its spores to simulated gastric conditions and for its antioxidant, antimicrobial, and antibiofilm activities. Strain A1 combines a safety profile with functional probiotic traits and is therefore a strong candidate for further *in vivo* evaluation and future development as a next-generation probiotic.

## 1. Introduction

The search for new probiotic strains with specific functional potential has enormously increased in recent years due to the need to develop alternatives to antibiotics [1] and novel natural health-promoting strategies [2,3]. Modulation of the immune system, production of antioxidants and antimicrobials, protection of the intestinal barrier integrity, and reduction in pathogen colonization are among the most relevant properties that have attracted interest in probiotic bacteria [2,3,4]. Several studies have focused on bacterial strains derived from the intestinal microbiota of healthy individuals as a source of potential probiotics. Indeed, such bacterial strains are potentially interesting candidates for probiotic applications because of their origin and functional traits, including resistance to acid and bile salts, colonization ability, and immune modulation potential [5,6].

Among members of the intestinal microbiota with potential probiotic properties, species of the genus *Bacillus* have attracted attention for their ability to form spores [7]. Spore formers are ubiquitous in nature and are common inhabitants of the animal gut [8,9]. Several spore-forming strains of the *Bacillus* genus are currently present on the probiotic market, and in all cases, the commercial formulation contains spores of one or more species [10]. Spores are extremely stable cell forms, resistant to water and nutrient deprivation, and to conditions of high temperature and low pH. Therefore, a commercial product containing spores is generally characterized by a very long shelf-life without the need for refrigerated storage [10]. Ingested spores of *B. subtilis* have been shown to safely cross the gastric barrier, germinate, and proliferate in the intestine, where the probiotic effects are exerted by the germination-derived cells [11,12,13]. Some *Bacillus* strains have demonstrated multiple functional properties relevant to probiotics, including anti-inflammatory, antioxidant, and antimicrobial effects, suggesting diverse mechanisms for health promotion [14]. For some strains, pre-clinical studies in animal models have indicated *in vivo* beneficial effects, including the reduction of diet-induced inflammation, restoration of a functional epithelial barrier, and the prevention of metabolic and cognitive disorders [15,16]. The beneficial effects of ingested spores have also been associated with the ability of spores to modulate the composition of the gut microbiota and favor the presence of beneficial bacteria, such as members of the *Akkermansia muciniphila* species and the *Bacteroidetes* genus [17].

The efficacy of new probiotic strains for human use has to be evaluated *in vivo* in pre-clinical studies and then, eventually, in human trials. However, it is essential to validate the functional properties of the newly isolated strains before any animal test. In this regard, bioinformatics tools and *in vitro* characterization have proven to be invaluable, enabling integrated, in-depth analysis of genetic and functional data, thereby supporting the selection of microorganisms with probiotic potential [18,19].

In this study, *Bacillus* strains were isolated from fecal samples collected from healthy children who had not received probiotic or antibiotic treatments. Two of these strains showed no hemolytic activity or antibiotic resistance and were identified as *B. spizizenii* (A1) and *Priestia megaterium* (formerly *B. megaterium*) (F6). These isolates were characterized for their probiotic potential through both *in vitro* and *in silico* analyses, and consequently, strain A1 is proposed as the most promising probiotic candidate.

## 2. Results and Discussion

### 2.1. Preliminary Characterization of Spore-Forming Strains from Fecal Samples

A total of 34 spore-forming strains were isolated from human fecal samples (Section 3.1). All strains were able to form spores in DS medium at 37 °C in aerobic conditions and were tentatively considered as belonging to the *Bacillus* genus. All strains were analyzed for their hemolytic activity and resistance to antibiotics. On sheep blood agar plates, 14 strains were unable to induce hemolysis and were therefore indicated as gamma-hemolytic, while the remaining 20 strains induced total hemolysis and were then indicated as beta-hemolytic (Supplementary Table S1). By using a panel of eight antibiotics considered relevant for members of the *Bacillus* genus [20], 17 strains resulted resistant only to streptomycin, one only to vancomycin, one only to kanamycin, and 13 to more than one antibiotic (Supplementary Table S2). Only two strains, A1 and F6, were sensitive to all tested antibiotics, showing minimum inhibitory concentration (MIC) values far below the respective cutoff points (highlighted in Supplementary Table S2).

These two strains, which were also gamma-hemolytic (unable to induce hemolysis) (Supplementary Table S1), were therefore considered potentially useful as probiotics and selected for further analysis.

### 2.2. Genome Sequence and Phylogenetic Analysis

Whole-genome sequence of A1 and F6 strains was obtained with a coverage of ~129 and 67, respectively. Strain A1 presented an assembly composed of 25 contigs, with an N50 value of 297,467 bp and an L50 of 5, indicating that half of the genome was contained within the five largest contigs. The total genome size was 4.0 Mb, with a GC content of 43.92%. CheckM analysis demonstrated 99.99% completeness and 0.39% contamination. A total of 3997 coding sequences (CDSs), 67 tRNA genes, and 17 rRNA genes were identified (Table 1). The genome of strain F6 consisted of 51 contigs, with an N50 value of 328,388 bp and an L50 of 7. F6 genome is approximately 5.85 Mb with a GC content of 37.45%. CheckM analysis indicated 100.0% completeness and 0.9% contamination. A number of CDSs of 6050 with 96 tRNA genes and 23 rRNA genes (Table 1).

**Figure 1:**
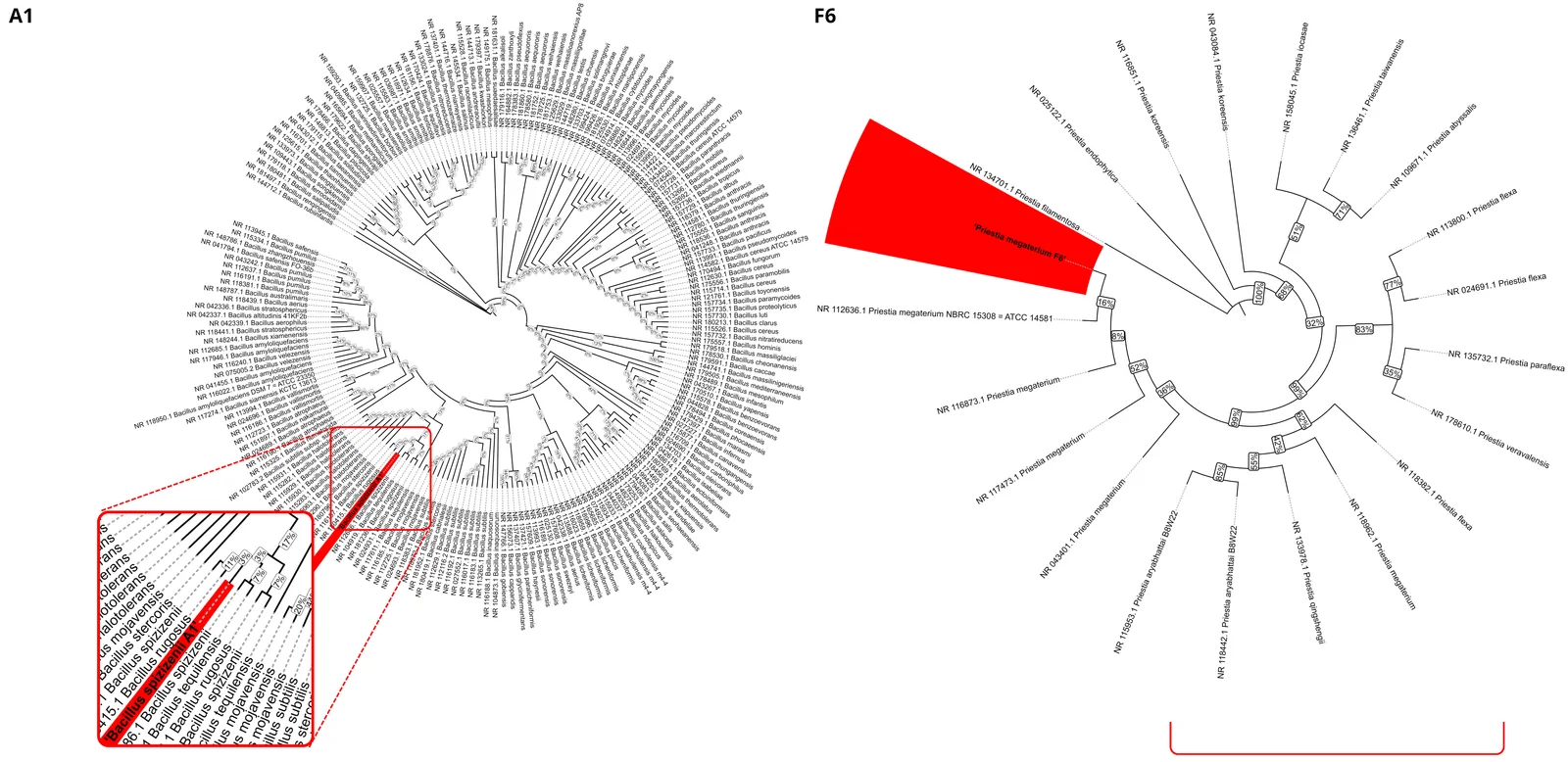
Maximum-likelihood phylogenetic inference based on 16S rRNA gene sequence alignments for *B. spizizenii* A1 and *P. megaterium* F6, illustrating evolutionary relationships and taxonomic placement within their respective generic clades.

A preliminary taxonomic classification of A1 and F6 based on the 16S rRNA gene sequences identified A1 and F6 as belonging to the *Bacillus spizizenii* and *Priestia megaterium* species, respectively, and, based on that, phylogenetic trees were constructed. As shown in Figure 1, strain A1 (Figure 1) clustered with species of the *Bacillus subtilis* complex, confirming its identification and indicating high genetic similarity with closely related strains, while remaining clearly separated from other groups. Similarly, strain F6 (Figure 1) occupied a central position, clustering with reference strains of the *P. megaterium* species, confirming its taxonomic identification and showing high genetic proximity to lineages such as ATCC 14581 and NBRC 15308. In contrast, *Priestia aryabhattai* strains form a distinct clade, further supporting the differentiation between these species. The species assignment was further confirmed by determining the Average Nucleotide Identity (ANI) and digital DNA–DNA hybridization (dDDH) values, which compare genomic similarity between the isolates and reference genomes (Table 2).

*Bacillus spizizenii* was originally classified as a subspecies of *B. subtilis* (*B. subtilis* subsp. *spizizenii*), but it is now considered a separate species due to accessory genome segments not found in the typical *B. subtilis* strains. Both *B. spizizenii* and *P. megaterium* (formerly *B. megaterium*) are generally considered safe and have a QPS (Qualified Presumption of Safety) status, provided they do not harbor acquired antimicrobial resistance genes and do not produce toxins [21].

### 2.3. Genomic Characterization of Strains B. spizizenii A1 and P. megaterium F6

The PATO predictive tool, a machine learning-supervised tool specifically designed to predict probiotic activity in microorganisms [18], assigned a high score to both strains, with scores of 95% for A1 and 78% for F6. Although the PATO predictive tool was originally designed for probiotics of the *Lactobacillus* genus, a high score could be considered as a preliminary indication of the probiotic potential. The higher score of strain A1 probably reflects a genomic signature highly consistent with established probiotic references.

A pangenomic analysis was performed comparing A1 and F6 genomes with those of 72 strains of *B. spizizenii* and 479 strains of *P. megaterium*, respectively. These strains were selected as the most genetically similar to A1 and F6. The analysis of the A1-*B. spizizenii* pangenome identified 7427 gene clusters, of which 4182 correspond to core genes (56.3%) and 3245 to variable fractions (cloud genes, 43.7%), with no shell genes or soft core genes identified (Table 3). In the pangenome of F6-*P. megaterium* strains, a substantial expansion is observed, with a total of 51,979 gene clusters, of which 6603 correspond to core genes (12.7%) and 45,376 to the variable fraction (cloud genes, 87.3%), also with no representatives in the intermediate categories of shell genes and soft core genes (Table 3).

**Figure 2:**
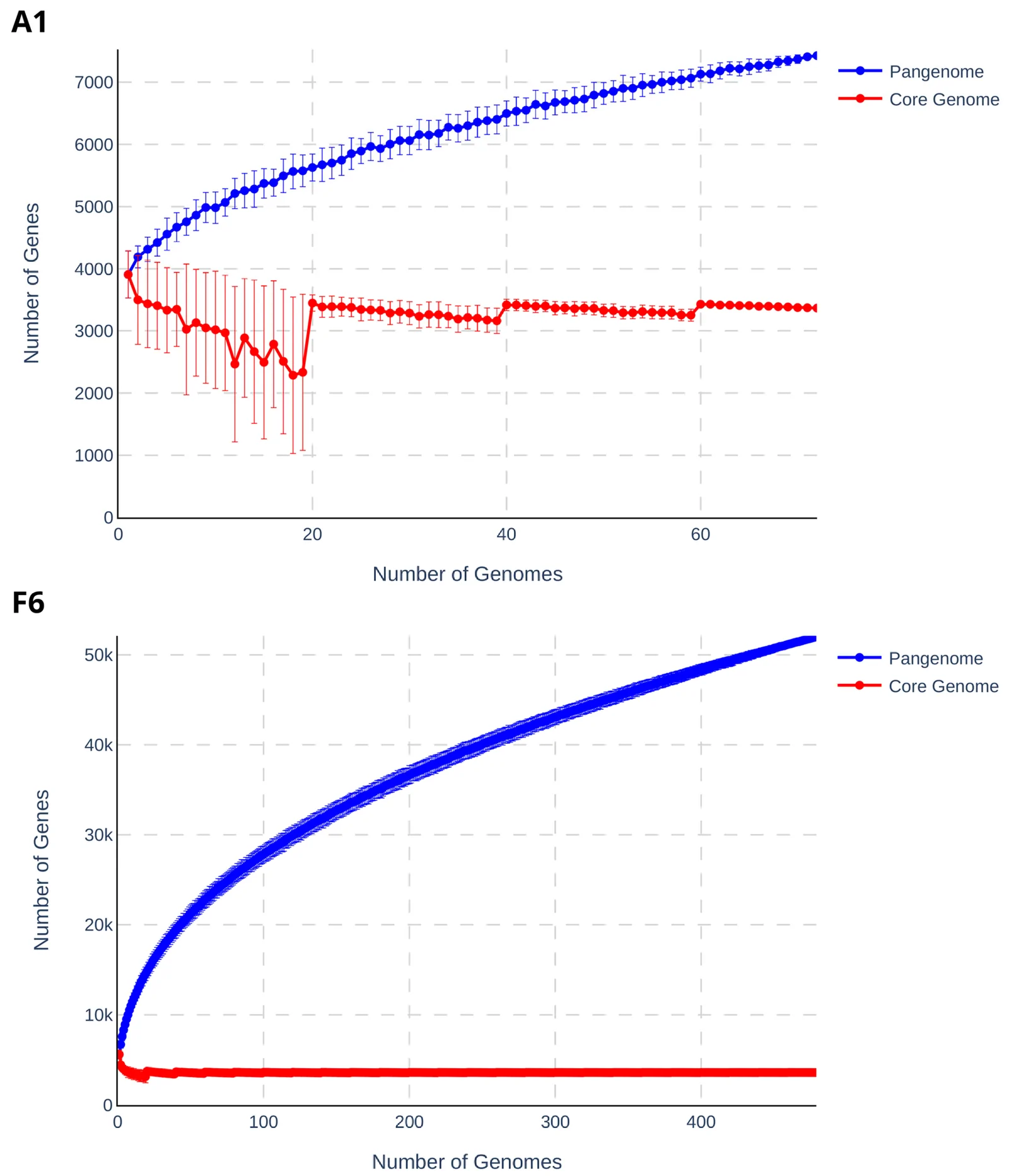
Pangenome and core genome accumulation curves for strain A1 and F6. The number of pangenome gene clusters (blue) and core genome gene clusters (red) were plotted against the number of genomes included in the analysis for the two distinct datasets.

The pangenome analysis revealed that both strains possess an open genomic repertoire, characterized by a continuous expansion of gene content and high genetic diversity, suggesting considerable adaptive potential [22]. From ecological and biotechnological perspectives, this pattern is particularly relevant, as open pangenomes are generally associated with greater genomic flexibility, enabling adaptation to diverse environmental selective pressures. In both panels of Figure 2, the pangenome exhibited continuous expansion as new genomes were incorporated, reinforcing the notion of high genetic diversity and ongoing acquisition of novel genes.

For strain F6, the core genome remained stable from the early stages of the accumulation curve, suggesting a conserved set of essential genes shared among all evaluated genomes. This stability aligns with the findings of Hyun and Palsson [23], who demonstrated that, although core genome composition may vary among species, the functional roles of these genes tend to remain conserved. In contrast, strain A1 exhibited a distinct pattern, with a pronounced reduction in core genome size during the initial stages of the accumulation curve, followed by stabilization at approximately 3000–3500 genes. However, these divergent patterns must be interpreted with caution. Rather than purely reflecting underlying evolutionary dynamics, they could be influenced by methodological variations, including the substantially different dataset sizes (72 versus 479 genomes), as well as potential disparities in genome assembly quality and species/strain sampling diversity. Additionally, the absence of genes within the soft-core and shell fractions in both strains suggests a bimodal pangenome distribution, a pattern that may stem both from distinct selective pressures acting on accessory genes and from technical limitations related to dataset size and diversity [24,25].

A further genomic characterization of the two probiotic candidates was focused on genes coding for potential virulence factors or involved in the synthesis of secondary metabolites. Table 4 reports the genes of A1 and F6 with the highest score as potential virulence factors, identified by using ABRicate against the VFDB (https://github.com/tseemann/abricate (accessed on 23 November 2025)). Four and eight genes were identified in the A1 and F6 chromosomes, respectively. These genes code for stress response proteins (*clpP* and *clpC*), the elongation factor Tu (*tufA*), and CapB and CapC (*capB, capC*), two enzymes needed for poly-gamma-glutammate (PGA) biosynthesis. While A1 chromosome contains only a *capC* homolog, F6 carries two copies of the *capBC* genes (Table 4). PGA is an anionic, water-soluble, non-toxic, and biodegradable poly-aminoacid composed of D- and L-glutamic acid residues, produced and secreted by strains of various *Bacillus* species, including *B. subtilis, B. licheniformis,* and *B. amyloliquefaciens* [26]. Pathogenic strains of *B. anthracis* also produce PGA that remains anchored to the membrane and is a critical virulence factor, essential for the bacteria to evade the host immune system. However, in *B. anthracis,* the products of the entire five-gene operon *capBCADE* (carried by the pOX2 plasmid) are needed to produce and anchor PGA to the membrane [27]. The lack of the entire *cap* operon on A1 and F6 chromosomes makes it extremely unlikely that the presence of only *capC* (A1) or *capBC* (F6) may represent a health risk.

Table 5 reports the *in silico* prediction of the gene clusters and of the secondary metabolites potentially produced by A1 and F6, obtained using the antiSMASH tool (http://antismash.secondarymetabolites.org). Strain A1 potentially codes for the siderophore bacillibactin and the iron chelator pulcherriminic acid, which are indirect antimicrobials, and for molecules with direct antimicrobial activity. These are the ribosomally produced subtilin and subtilosin A, respectively active against *Staphylococcus aureus* and *Listeria monocytogenes,* and the non-ribosomal lipopeptides surfactin, bacillaene and mycosubtilin. While surfactin has anti-*S. aureus* activity, bacillaene has antimicrobial activity against a broad spectrum of bacteria, and mycosubtilin belongs to the iturin family and has antifungal activity. Those lipoproteins are potentially toxic for human cells at high concentrations, acting primarily on cell membranes. However, the observed lack of hemolytic activity of strain A1 (Table S1) made unlikely such possibility. Such genome-based predictions will need to be experimentally validated to conclude that strain A1 produces antimicrobial molecules. Strain F6 does not code for molecules with direct antimicrobial activity but for several siderophores with indirect antimicrobial activity (Table 5). Such results indicate only the genomic potential that needs to be experimentally validated.

The genomes of strain A1 and F6 were screened for the presence of antimicrobial-resistance genes by using the CARD database. As shown in Supplementary Table S4, several genes potentially coding for efflux pumps and other antibiotic-resistance determinants were identified. Some of those determinants could cause resistance to aminoglycosides, macrolides and tetracycline. However, both strains were sensitive to the amynoglycoside gentamicin, to the macrolide erythromycin and to tetracycline (Table S2). Therefore, the genome-based predicted presence of potential resistance determinants does not affect the response of A1 or F6 to the panel of antibiotics considered relevant by the EFSA [21].

### 2.4. Physiological Characterization of B. spizizenii A1 and P. megaterium F6

To characterize *in vitro* the probiotic potential of A1 and F6, a series of probiotic-related phenotypes was analyzed. Survival to gastric and/or intestinal conditions is clearly a parameter for a potential probiotic strain. Both cells and spores of the two strains were independently incubated for 1 h in simulated gastric fluid (SGF) or in simulated intestinal fluid (SIF) or sequentially in SGF and then in SIF (SGF + SIF). After the treatment, cells and spores were serially diluted, and the colony forming units (CFUs) determined on LB plates. As summarized in Table 6, A1 spores were resistant to all three treatments (Log reduction < 1), while an about 2-log decrease in CFU/mL was observed when A1 cells were treated. For strain F6, spores were slightly sensitive to SGF and SGF + SIF (Log reduction > 1), while a 2 to 3-log decrease in CFU/mL was observed when cells were treated (Table 6).

Biofilm formation by A1 and F6 cells was evaluated in rich (LB), minimal (S7), or sporulation-inducing (DS) media, in comparison with cells of other strains of their respective group/species. A1 was compared with PY79, a lab collection strain [28], and MV24, an intestinal isolate strain [1] of *B. subtilis*, while F6 was compared with QM B1551, a lab collection strain [29], and with MV30, an intestinal isolate [1] of *P. megaterium*.

**Figure 3:**
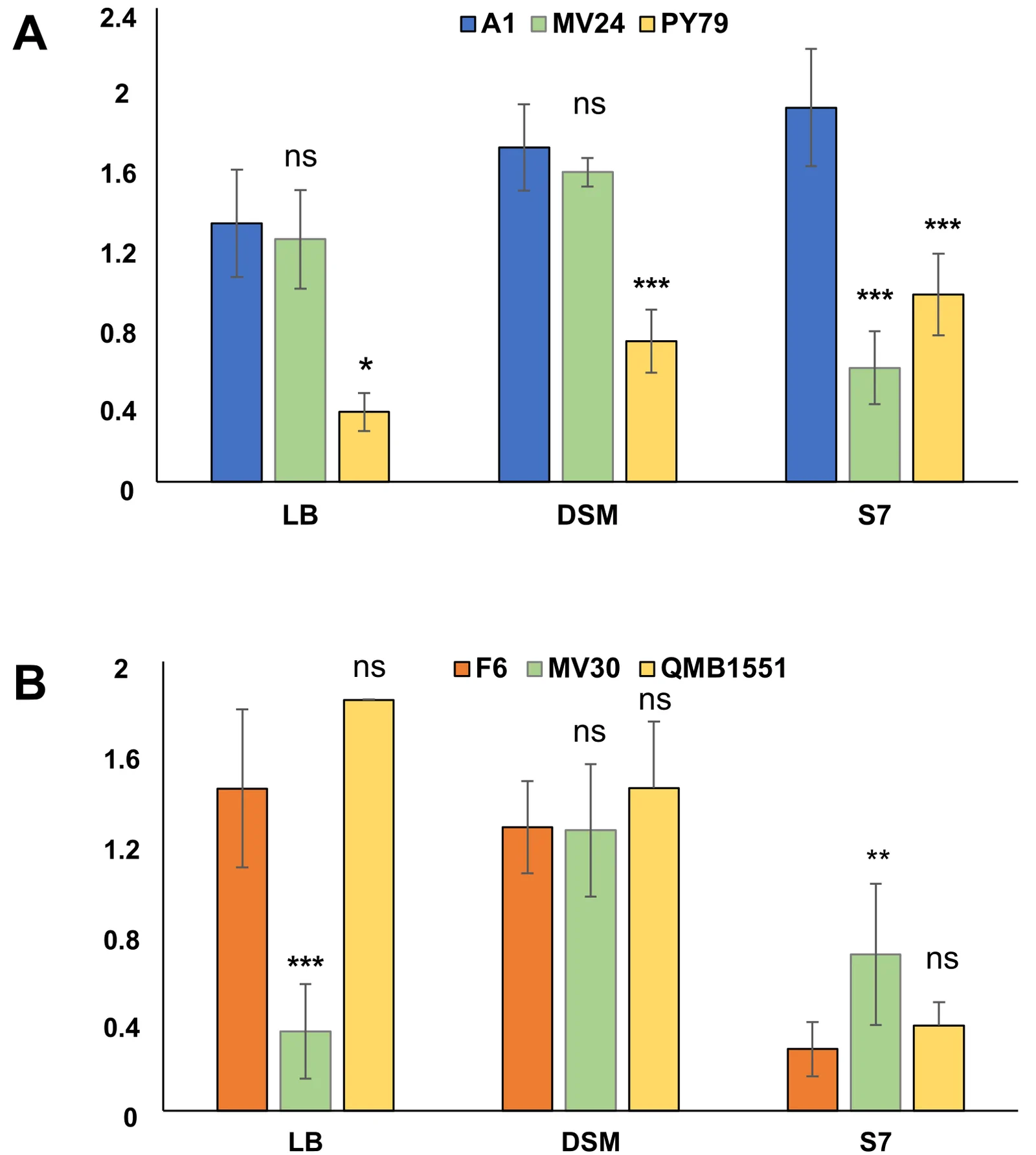
Biofilm formation of A1 and two other strains of the *B. subtilis* complex (**A**) and of F6 and two other strains belonging to the *P. megaterium* complex (**B**). Data are presented as mean + standard deviation (SD) from three independent biological replicates. Normality was assessed using the Shapiro–Wilk test, and homogeneity of variances was evaluated using Levene’s test. Differences among groups were analyzed by one-way analysis of variance (ANOVA), followed by Tukey’s honestly significant difference (HSD) post hoc test for multiple comparisons. Statistical analyses were performed using R (version 4.4.0), and differences were considered statistically significant at *p* < 0.05. ns: not significant, * *p* < 0.05, ** *p* < 0.01, *** *p* < 0.001 vs. control group (A1 or F6).

As shown in Figure 3A, A1 produced a similar amount of biofilm compared to MV24 in both LB and DSM, but significantly more biofilm in S7 medium. Furthermore, A1 formed more biofilm than PY79 across all three tested media. F6 cells (Figure 3B) produced more biofilm than MV30 only when grown in LB, while showing no significant differences in biofilm formation across all three media compared to the *P. megaterium* type strain QM B1551.

**Figure 4:**
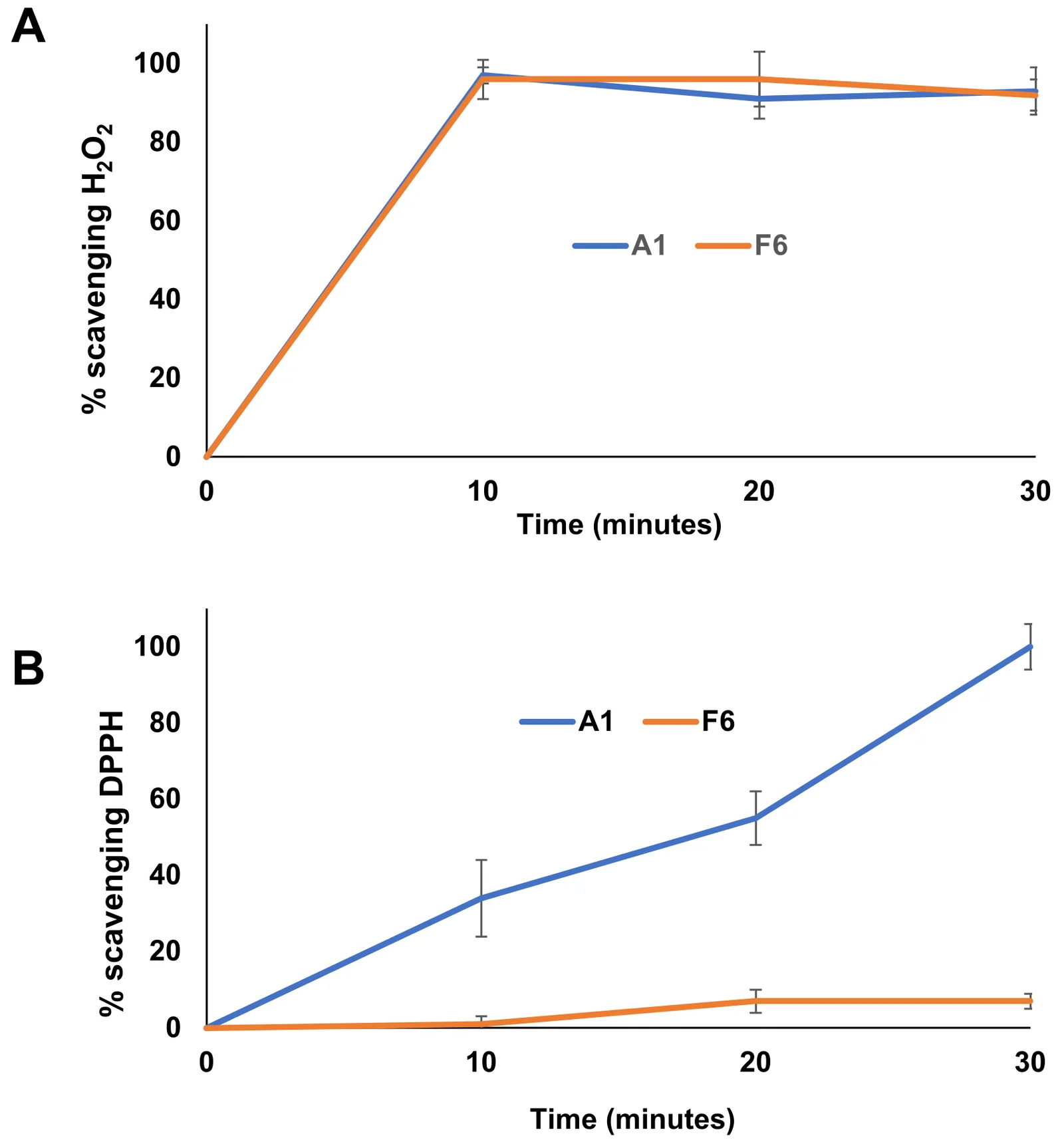
Antioxidant activities by A1 and F6 supernatant. Scavenging of H_2_O_2_ (**A**) or DPPH (**B**) were assayed.

Cell-free supernatantss of strains A1 and F6 were tested for the production of molecules with antioxidant activity by assaying the scavenging activity against hydrogen peroxide (H_2_O_2_) or free radicals (by the α,α-diphenyl-b-picrylhydrazyl (DPPH) method), as previously described [30]. As reported in Figure 4A, both strains showed a strong H_2_O_2_-scavenging activity with an almost total elimination of the H_2_O_2_. A1 cells also showed a strong scavenging activity against free radicals that was, instead, not present in F6 cells (Figure 4B).

To evaluate the production of antimicrobial molecules, A1 and F6 cells were grown in minimal (S7) or rich (LB) medium. The culture supernatant was filter-sterilized and tested against a panel of target microorganisms by agar diffusion assays. As reported in Table 7, strain F6 did not produce molecules active against any of the tested microorganisms, while strain A1 grown in LB medium, produced molecules active against strains of *Enterococcus faecalis*, *Staphylococcus aureus, Priestia megaterium*, and *Listeria monocytogenes*. When grown in S7 medium, A1 only produced molecule(s) active against *L. monocytogenes* (Table 7). The results in Table 7 are consistent with the predicted antimicrobial potential reported in Table 5, with F6 predicted to be unable to produce direct antimicrobials and A1 able to produce molecules active against *S. aureus* (subtilin, surfactin) and *L. monocytogenes* (subtilosin A). The lack of the broad-spectrum antimicrobial activity expected from the predicted synthesis of bacillaene could be due to the well-known instability of the molecule [31], while the lack of antifungal activity expected based on the potential production of mycosubtilin could be due to low expression of the non-ribosomal peptide synthase gene under the laboratory conditions used. Such hypotheses will need to be experimentally verified in a future study.

### 2.5. A1 and F6 Cells Produce a Molecule with Antibiofilm Activity

**Figure 5:**
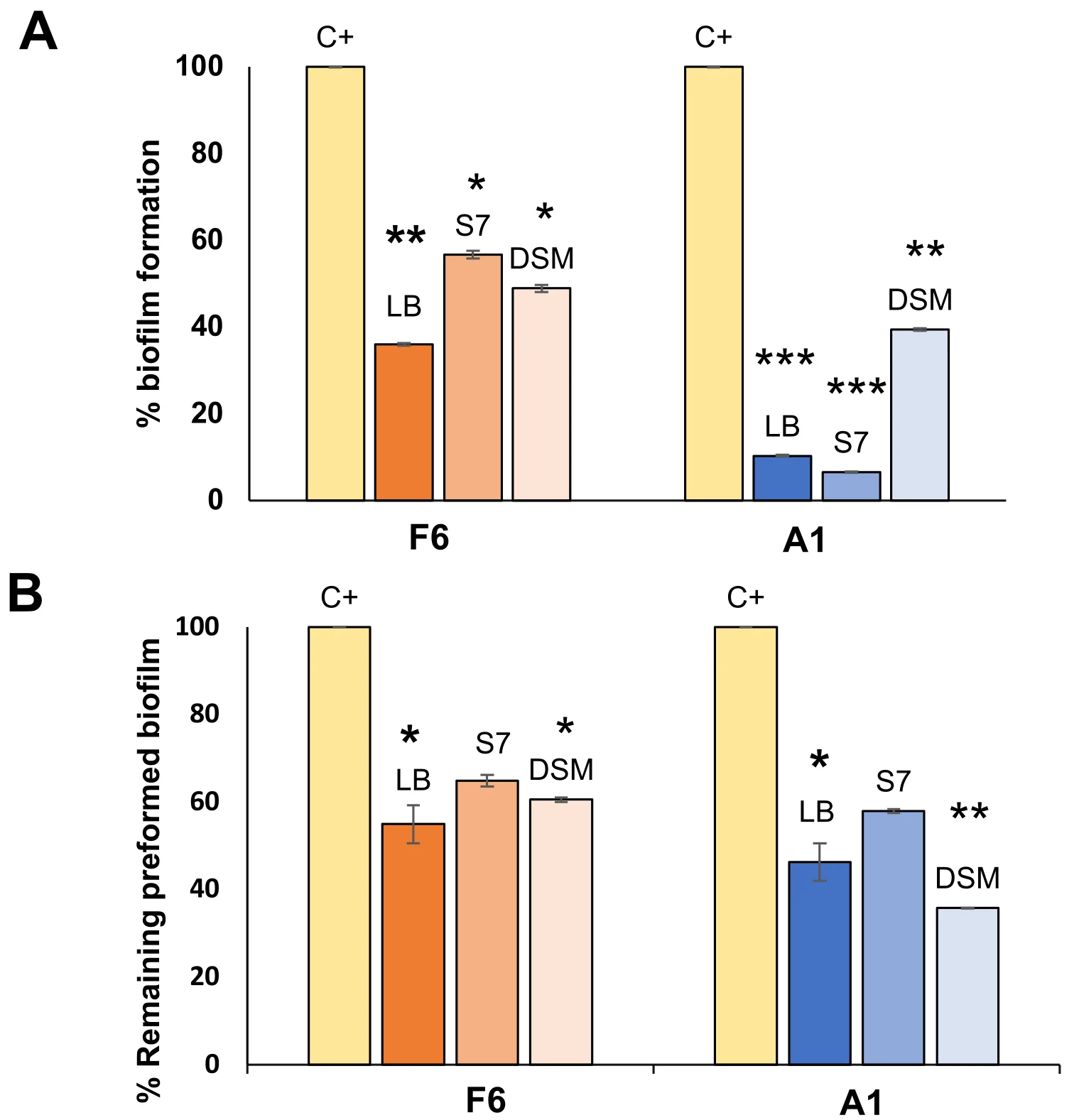
Antibiofilm activity of cell-free supernatants A1 and F6 cells grown in different media. The inhibition of biofilm formation (**A**) and the reduction in pre-formed biofilm (**B**) were assayed. Total biofilm formed by *M. smegmatis* was reported as 100% (C+). Data are presented as mean ± SD, *n* = 3 (biological triplicates); statistical significance was determined using Student’s *t*-test. * *p* < 0.05, ** *p* < 0.01, *** *p* < 0.001 vs. control group.

The cell-free supernatants of A1 and F6 cells grown in different media were tested for their antibiofilm activity against a biofilm-producing strain of *Mycobacterium smegmatis* [30]. Both strains produced molecules able to both reduce biofilm formation (Figure 5A) and partially disassemble the pre-formed biofilms (Figure 5B). The antibiofilm activity observed with F6 supernatants was not significantly affected by the growth medium utilized, while that observed with A1 supernatants was stronger when cells were grown in rich (LB) or minimal (S7) medium than in sporulation-inducing (DS) medium (Figure 5). While the two strains performed similarly against pre-formed biofilms (Figure 5B), A1 appeared more active than F6 in reducing biofilm formation, at least when grown in LB or S7 medium (Figure 5A).

Since the cell-free supernatants of strains A1 and F6 did not affect the growth of *M. smegmatis* (Table 7), the results reported in Figure 5 may indicate that molecules secreted by A1 or F6 cells directly act on biofilm production and structure.

**Figure 6:**
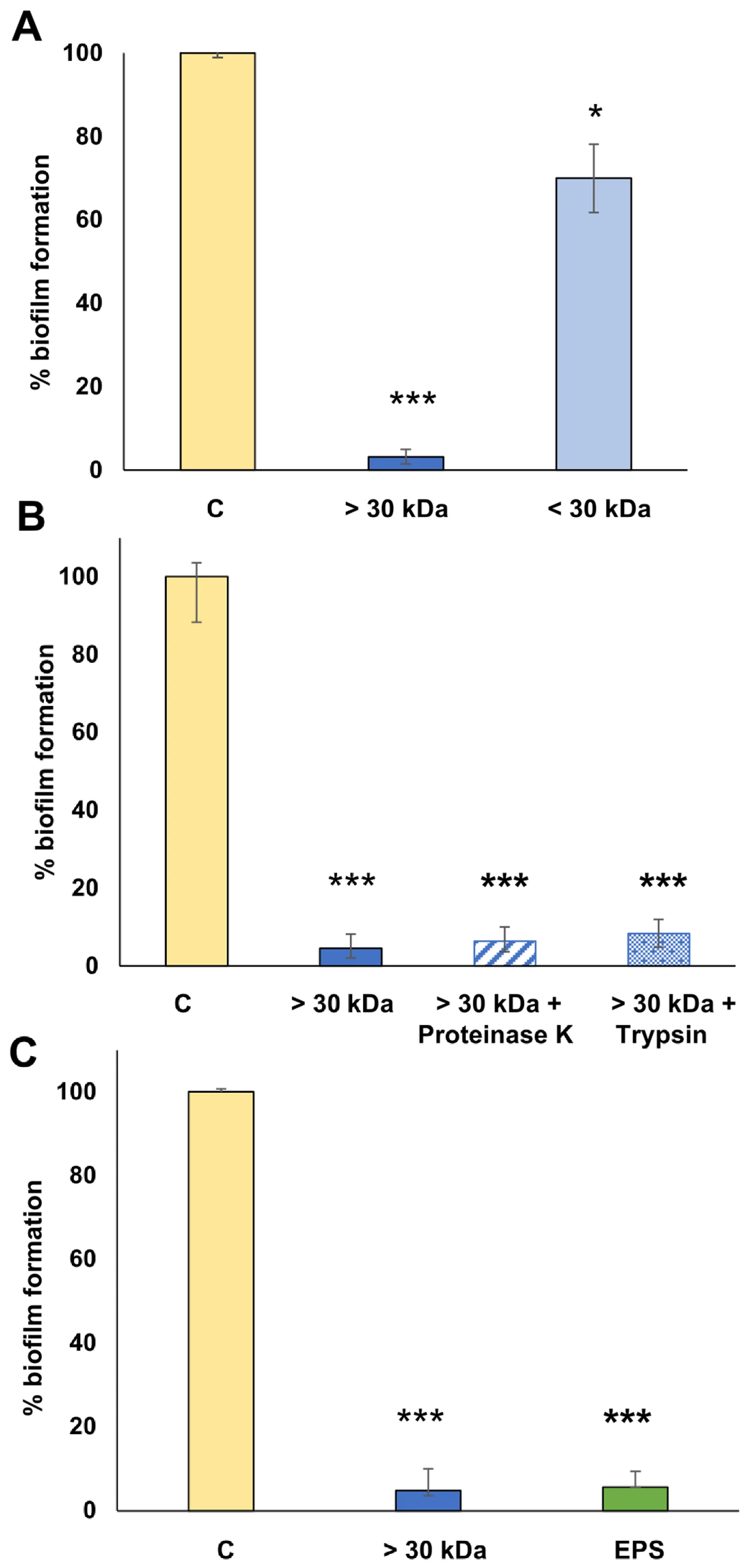
Antibiofilm activity of (**A**) fractionated cell-free supernatant of the A1 strain grown in LB medium, (**B**) the active fraction (>30 kDa) after protease treatments and (**C**) the purified EPS produced by the A1 strain. Data are presented as mean ± SD, n = 3; statistical significance was determined using Student’s *t*-test. * *p* < 0.05, *** *p* < 0.001 vs. control group.

To further characterize the antibiofilm activity, attention was focused on the most active strain (A1) grown in LB medium and tested against biofilm formation. The A1 cell-free supernatant was size-fractionated, and different fractions were tested for activity. All fractions containing molecules smaller than 30 kDa showed minimal activity in reducing biofilm formation, whereas this activity was strong in the >30 kDa fraction (Figure 6A). Treatment of the active >30 kDa fraction with trypsin or proteinase K did not affect the antibiofilm activity (Figure 6B), suggesting the non-proteinaceous nature of the antibiofilm molecule produced by A1 cells.

Several strains of the *B. subtilis* complex are known to produce molecules with antibiofilm activity [32]. Such activity has been so far associated with either lipopeptides of the surfactin, iturin, fengycin families, or with quorum quenching (QQ) factors, enzymes or exopolysaccharides (EPS) [32]. The size (>30 kDa) and the non-proteinaceous nature of the antibiofilm molecule of A1 suggested that lipopeptides, QQ and enzymes are most likely not involved in the observed activity and pointed to the EPS of A1 as a potential antibiofilm molecule. To evaluate the possible role of the EPS of strain A1 in the antibiofilm activity, the EPS was extracted as previously reported [32], and tested. As shown in Figure 6C, the purified EPS showed an antibiofilm activity similar to that observed with the >30 kDa fraction of the cell-free supernatant, suggesting that the EPS-enriched fraction of strain A1 is responsible for the observed effect.

## 3. Materials and Methods

### 3.1. Strain Isolation

Bacteria characterized in this study are part of a strain collection and were previously isolated from fecal samples collected from children aged 4 to 7 years who had not received antibiotics or probiotic treatments within the three months preceding sampling. Parents of the five children enrolled in the study provided that information by filling in a structured questionnaire with health information and antibiotic/probiotic use by their children. Approximately 1 g of feces was homogenized in sterile phosphate-buffered saline (PBS), subjected to heat treatment at 80 °C for 10 min and plated on Difco Sporulation Medium (DSM; for 1 L: 8 g/L Nutrient Broth, 1 g/L KCl, 1 mM MgSO_4_, 1 mM Ca(NO_3_)_2_, 10 mM MnCl_2_, 1 mM FeSO_4_, Sigma-Aldrich, Steinheim, Germany) to promote the growth of spore-forming bacteria. Plates were incubated aerobically at 37 °C for 24–48 h, then individual colonies were selected and isolated as previously described [8]. Potential siblings were defined as colonies with very similar morphology originating from the same sample. A total of 34 bacterial isolates capable of sporulation in DSM in aerobic conditions were obtained and stored at −80 °C. Those 34 strains were streaked from the −80 °C stabs and used for the described experiments.

### 3.2. Hemolytic Activity and Antibiotic Susceptibility

Hemolytic activity was assessed by spotting 5 µL of actively growing cultures onto Columbia agar plates supplemented with 5% defibrinated sheep blood, followed by incubation at 37 °C for 24 h. The presence of a clear halo surrounding the colonies was considered indicative of hemolytic activity. Hemolytic activity was evaluated in three independent assays, and positive and negative control strains were included for the evaluation of γ- and β-hemolytic phenotypes.

All strains were evaluated for antibiotic susceptibility by determining the minimum inhibitory concentration (MIC), as described in the European Food Safety Authority documentation [33]. Bacterial suspensions standardized to an optical density of 0.1 at 600 nm (OD_600_) were spread onto Tryptone Yeast Extract Broth (TY) agar plates, and MIC Test Strips (Liofilchem^®^, Roseto, Italy) impregnated with the following antibiotics were placed on the surface. The eight antibiotics tested, along with their concentration ranges and EFSA cutoff values, are summarized in Table S2. Plates were incubated overnight at 37 °C, and the MIC was determined at the point of intersection between the inhibition zone and the antibiotic gradient on the strip. Each MIC determination was performed in three independent assays. Strains were classified as resistant (R) when MIC values exceeded the microbiological cutoff values established by the EFSA Panel on Additives and Products or Substances used in Animal Feed [33].

### 3.3. Antimicrobial Activity, Motility, and Biofilm-Related Assays

Antimicrobial activity was determined using cell-free supernatants from cultures grown overnight in Tryptone Yeast Extract Broth (TY) or for 48 h in Minimal Medium (S7, 50 mM morpholine-propane-sulfonic acid (MOPS), adjusted to pH 7.0 with KOH, 10 mM (NH_4_)_2_SO_4_, 5 mM potassium phosphate (pH 7.0), 2 mM MgCl_2_, 0.9 mM CaCl_2_, 50 mM MnCl_2_, 5 mM FeCl_3_, 10 mM ZnCl_2_, 2 mM thiamine hydrochloride, 20 mM sodium glutamate, 1% glucose, 0.1 mg/mL phenylalanine, and 0.1 mg/mL tryptophan). The pH of the cell-free supernatants was measured before antimicrobial activity assays and, when necessary, adjusted to neutral pH to exclude the contribution of acidity to bacterial growth inhibition. Indicator strains used for the antimicrobial assay are listed in Table 7. Overnight cultures of each strain were grown in LB medium and standardized to an OD_600_ of approximately 0.1 before inoculation onto LB agar plates. Cell-free supernatants (30 µL) were spotted onto the agar plates previously inoculated with the indicator strains. Plates were incubated overnight at 37 °C, and the presence and diameter of inhibition zones were recorded.

Biofilm formation was assessed in LB, DS and S7 media by inoculating overnight cultures standardized to an OD_600_ of 0.1 and incubating for 48 h. Cells forming a solid layer at the liquid–air interface were considered biofilm producers [34].

Antibiofilm activity was evaluated using cell-free supernatants, which were fractionated using Amicon Ultra centrifugal filter units with a 30 kDa molecular weight cutoff membrane (Millipore) according to the manufacturer’s instructions. The <30 kDa fraction corresponded to the filtrate, whereas the >30 kDa fraction corresponded to the retained material. After fractionation, both fractions were recovered and volumes were restored to the original starting volume before antimicrobial activity assays.

*Mycobacterium smegmatis* was cultured in 24-well plates containing Sauton medium (0.05% KH_2_PO_4_, 0.05% MgSO_4_·7H_2_O, 0.2% citric acid, 0.005% ferric ammonium citrate, 6% glycerol, 0.4% asparagine, 0.05% Tween 80, pH 7.4) under static conditions at 37 °C for 72 h. For pre-exposure experiments, cell-free supernatants from A1 and F6 were added to the wells together with the target bacterial strains, whereas for post-exposure experiments, the supernatant was added to pre-formed biofilms and incubated for 24 h at 37 °C. Biofilm production was quantitatively assessed using the crystal violet assay, as previously described by Vittoria et al. [35]. Briefly, attached biofilm biomass was stained with crystal violet and quantified by measuring absorbance. Data were normalized to total growth measured by OD_590_ according to the formula: OD_590_ after washes/OD_590_ before washes. All experiments were performed using three independent biological replicates.

### 3.4. Spore Preparation, Purification, and Simulated Gastric and Intestinal Fluid Treatments

Sporulation was induced using the exhaustion method [36]. After 30 h of growth in DSM at 37 °C under vigorous shaking, spores from *B. spizizenii* A1 and *P. megaterium* F6 were collected, washed three times with distilled water, and incubated overnight in distilled water at 4 °C to lyse residual sporangial cells, as previously described [37]. Spore concentrations were determined by direct counting using a Bürker chamber [Sigma, St. Louis, MO, USA (BR719505)] under an optical microscope (Olympus BH-2 equipped with a 40× objective).

Purified spores (approximately 10^8^ spores per assay) were exposed to simulated gastric fluid (SGF; 1 mg/mL pepsin from porcine stomach mucosa in 10 mM HCl, pH 2.0) or simulated intestinal fluid (SIF; 1 mg/mL pancreatin from porcine pancreas supplemented with 0.2% bile salts [50% sodium cholate and 50% sodium deoxycholate], pH 6.8) for 1 h at 37 °C. After treatment, samples were neutralized, serially diluted, and plated for viable spore enumeration. Each treatment was performed using three independent biological replicates.

### 3.5. Antioxidant Activity Assays

As previously reported [1] the hydrogen peroxide scavenging activity of A1 and F6 strains was determined by measuring the decrease in absorbance at 240 nm. Briefly, cell-free supernatants (10% *v*/*v* in LB broth) of both strains were incubated at room temperature in 1 mL of a 0.036% (*w*/*w*) $H_{2}O_{2}$ solution prepared in 50 mM potassium phosphate buffer (pH 7.0). After 30 min, the residual $H_{2}O_{2}$ concentration in the supernatant was quantified by measuring the absorbance at 240 nm. The percentage of removed hydrogen peroxide was calculated using the following equation:

$$H_{2}O_{2}\;\mathrm{removed}\;\left(\%\right)=\left(1-\frac{A_{\mathrm{sample}}}{A_{\mathrm{control}}}\right)\times 100$$

The potential antioxidant activity of the A1 and F6 cells was also evaluated using the 2,2-diphenyl-1-picrylhydrazyl (DPPH) free radical scavenging method. Cell-free supernatants (10% *v*/*v* in LB broth) from both strains were mixed in a final volume of 1 mL of 50 mM citrate-phosphate buffer (pH 7) containing 0.1 mM freshly prepared DPPH (initial absorbance ≤1.0). The reaction mixtures were incubated for 30 min at room temperature in the dark. The scavenging activity was then determined by measuring the absorbance at 515 nm and calculated according to the formula, as previously reported [1]:

$$\mathrm{DPPH}\;\mathrm{scavenging}\;\mathrm{activity}\;\left(\%\right)=\left(1-\frac{A_{\mathrm{sample}}}{A_{\mathrm{control}}}\right)\times 100$$

where A_sample_ represents the absorbance of the reaction mixture containing the bacterial cells, and A_control_ is the absorbance of the control DPPH solution.

### 3.6. EPS Extraction

A1 and F6 strains were grown with a starting OD_600nm_ of 0.1 in LB medium at 37 °C for 24 h. After growth, the cultures were centrifuged (at 9800× *g* for 20 min) and the supernatants were filtered and separated into two aliquots of identical volumes. One aliquot was kept as the cell-free supernatant, and the other one was subjected to ethanol precipitation as previously described [32] to obtain EPS. More specifically, two volumes of cold absolute ethanol were added to the filtered supernatant and the samples were incubated overnight at 4 °C. Subsequently, the aliquot was centrifuged at 5000× *g* for 30 min, and the precipitate was washed two times with cold absolute ethanol and resuspended in the same initial volume using hot water (80–90 °C) [32].

### 3.7. Whole-Genome Sequencing

Bacterial genomic DNA was extracted from exponentially growing cells as previously reported [38]. Whole-genome sequencing of strains A1 and F6 was performed using the Illumina NextSeq Sequencing System with paired-end reads (2 × 150 bp). Quality control was performed using FastQC (version 0.11.9) and Fastp (version 0.23.2). The expected post-processing sequencing depths were calculated to be 132X for strain A1 and 70.15X for strain F6. Genome assemblies were generated with SPAdes v3.14.0 [39] with default settings, and genome annotation was performed using NCBI PGAP (https://www.ncbi.nlm.nih.gov/refseq/annotation_prok/ (accessed on 28 October 2025)).

The whole-genome shotgun projects for *Priestia megaterium* strain F6 and *Bacillus spizizenii* strain A1 have been deposited at DDBJ/ENA/GenBank under BioProject accession number PRJNA1406710. The genome sequences are available under GenBank accession numbers JBTXTM000000000.1 and JBTXTN000000000.1, respectively, corresponding to BioSample accession numbers SAMN54800019 for strain F6 and SAMN54800018 for strain A1.

### 3.8. In Silico Prediction of Probiotic Activity

PATO (Probiotic Analysis Tool), version 1.0 (http://200.132.101.156:5001/) (accessed 13 January 2026) [18], with a 0.5 classification threshold was used to predict the probiotic potential of the selected strains.

### 3.9. Species-Level Taxonomy and Phylogenetic Tree Construction

Average Nucleotide Identity (ANI) analysis was performed with fastANI version 1.3 (https://github.com/ParBLiSS/FastANI, accessed on 5 August 2026), establishing scores ≥95% as indicative of species-level identification. Taxonomy was expanded via whole-genome analysis using the Type (Strain) Genome Server (TYGS) platform (https://tygs.dsmz.de/), accessed on 13 March 2026, serving as an *in silico* alternative to traditional DNA–DNA hybridization. Closely related type strains were identified through the MASH (version 2.3) algorithm for intergenomic relatedness approximation and BLAST (version 2.14.1) evaluation of 16S rDNA gene sequences. Digital DNA–DNA hybridization (dDDH) values and confidence intervals were computed with the Genome-to-Genome Distance Calculator (GGDC 4.0). Pairwise comparisons utilized the Genome BLAST Distance Phylogeny (GBDP) approach. Final dDDH derivation applied GBDP formula 4 (sum of identities in HSPs divided by overall HSP length) to guarantee calculation robustness across draft genomes. Taxonomic nomenclature and synonymy were validated against the List of Prokaryotic names with Standing in Nomenclature (LPSN). Phylogenetic tree construction for strains A1 and F6 utilized 16S rRNA gene sequences. Sequences were processed through the BLAST algorithm (NCBI) to identify homologous sequences, prioritizing targets by identity and alignment coverage [40]. MUSCLE [41] executed sequence alignment for evolutionary inference [42]. Ambiguously aligned positions were eliminated using trimAL [43], isolating phylogenetically informative regions [44]. Phylogenetic inference was performed using the multi-threaded, AVX-optimized distribution of RAxML (raxmlHPC-PTHREADS-AVX) executing across 16 threads. A rapid bootstrap analysis and a search for the best-scoring maximum-likelihood tree were conducted simultaneously. The General Time Reversible nucleotide substitution model incorporating a GAMMA distribution for among-site rate heterogeneity (GTRGAMMA) was applied. Random seeds for both the parsimony starting tree and the rapid bootstrap algorithm were set to 12345. Statistical branch support was calculated utilizing 1000 bootstrap replicates derived from the multiple sequence alignment file.

### 3.10. Pangenomic Analysis

The pangenomic analysis of strains A1 and F6 was performed using the Roary v3.13 software [45], employing GFF3 files as input. The genomes were initially annotated and standardized in this format to ensure compatibility with the software. In order to define the core genome, genes (core genes) present in at least 95% of the analyzed genomes were considered, with a minimum sequence similarity threshold of 70% for clustering into orthologous clusters [46]. Gene clustering was performed through similarity analyses with BLASTp and clustering algorithms, generating a gene presence/absence matrix that enabled the identification of different fractions of the pangenome, such as core genes, accessory genes (shell), and unique genes (cloud genes) [47]. Pangenome and core genome accumulation curves were generated utilizing a number of random genome-order permutations equal to the total number of genomes in each dataset (72 for A1, 479 for F6). The mean and standard deviation of gene counts were computed as measures of uncertainty for these distributions.

To define the pangenome partitions, the following frequency thresholds were utilized: Core: 95% ≤ strains ≤ 100%; Soft-core: 15% ≤ strains < 95%; Shell: 5% ≤ strains < 15%; and Cloud: 0% < strains < 5%. Duplicated or paralogous genes were split into separate clusters rather than merged, reflecting the absence of the ‘-s’ flag in the Roary configuration. Accession number of the reference genomes are reported in Supplementary Table S3.

### 3.11. Prediction of Production of Toxins, Virulence Factors, and Secondary Metabolites

The identification of potential virulence genes in the genomes of isolates A1 and F6 was performed using the ABRicate software (version 1.4.0) [48], with queries against the Virulence Factor Database (VFDB) and the CARD database (version 2025) [49]. The assembled genomes, in FASTA format, were subjected to ABRicate analysis to screen for genes associated with bacterial virulence factors through sequence similarity-based alignments. The VFDB, which compiles experimentally characterized virulence genes, was utilized for gene identification. Genes were considered present if they exhibited a minimum threshold of 70% sequence identity and 60% coverage [49,50].

In addition, secondary metabolite prediction was performed using antiSMASH (antibiotics and Secondary Metabolite Analysis Shell), version 8.0 (2025) [51]. This is a free and widely used online bioinformatics platform designed for the comprehensive identification of biosynthetic gene clusters (BGCs) in genomic sequences (https://antismash.secondarymetabolites.org/ (accessed on 21 July 2026)).

## 4. Conclusions

The screening of aerobic spore formers isolated from human fecal samples highlighted that only 2 out of 34 strains were non-hemolytic and not resistant (remaining below the tested MIC cutoffs) to a panel of antibiotics indicated as relevant for members of the *Bacillus* genus. The two selected strains, A1 and F6, were characterized using *in silico* and *in vitro* for their probiotic potential.

Both strains lacked known genomic determinants under the applied search criteria. The comparative analysis of the two strains indicated A1 as the most promising probiotic candidate. Such conclusion arises from several lines of evidence:

- The PATO predictive tool, a machine learning-supervised tool specifically designed to predict probiotic activity in microorganisms [48], assigned a higher probiotic score to A1 than to F6;
- The resistance to simulated gastric conditions was higher for A1 than F6 spores. This is relevant because most probiotics based on spore-forming species are administered as spores that have to safely cross the gastric barrier in order to germinate in the intestine and give rise to the vegetative cells responsible for the probiotic activity [10];
- While both A1 and F6 cell-free supernatants showed antioxidant activity and showed H_2_O_2_-scavenging activities, only A1 supernatant also showed scavenging activity against free radicals;
- A1 cells produce and secrete molecules active against cells of *Enterococcus faecalis*, *Staphylococcus aureus, Priestia megaterium*, and *Listeria monocytogenes* cells, while F6 does not produce antimicrobials;
- A1 cells are more active than F6 cells in inhibiting biofilm formation.

Altogether, the reported results point to A1 as a candidate probiotic strain that warrants a further evaluation in *in vivo* models to assess its beneficial effects.

## Figures and Tables

**Table 1:** General features of A1 and F6 genomes.

Isolate	Number of Contigs	N50	L50	Total Length	GC Content	Features		
						CDS	tRNA	rRNA
A1	25	297,467	5	4,001,935	43.92	3997	67	17
F6	51	328,388	7	5,853,006	37.45	6050	96	23

**Table 2:** Taxonomic classification.

Strain	ANI		dDDH	
	Closest Species	Similarity	Closest Species	Similarity
A1	*B. spizizenii* S1 (GenBank: GCF_048551475.1)	99.25%	*B. spizizenii* TU-B-10 (GenBank: GCF_000227465.1)	93.7%
F6	*P. megaterium* MV30 (GenBank: GCF_030767185.1)	99.98%	*P. megaterium* ATCC 14581 (GenBank: GCF_006094495.1)	93.6%

**Table 3:** Comparative pangenome partition analysis detailing the distribution of core, soft core, shell, and cloud gene clusters for *B. spizizenii* A1 and *P. megaterium* F6.

	A1	F6
Species	*Bacillus spizizenii*	*Priestia megaterium*
Total Number of Strains	72	479
Core genes	4182	6603
Soft core genes	0	0
Shell genes	0	0
Cloud genes	3245	45,376
Total pangenome	7427	51,979

**Table 4:** Potential virulence genes present on the chromosome of A1 or F6 strains.

Contig	Start	End	Coverage	Identity	VFDB Virulence Factor		
					Gene	Name	Category
**A1**							
JBTXTN010000004	113,252	113,818	94.64	73.46	*clpP*	ATP-dependent Clp protease proteolytic subunit	Stress survival (VFC0282)
JBTXTN010000004	268,733	269,182	99.78	71.62	*capC*	CapC involved in Poly-gamma-glutamate synthesis	Immune modulation (VFC0258)
JBTXTN010000010	2469	4826	95.05	71.42	*clpC*	endopeptidase Clp ATP-binding chain C	Stress survival (VFC0282)
JBTXTN010000010	31,778	32,968	99.58	73.24	*tufA*	elongation factor Tu	Adherence (VFC0001)
**F6**							
JBTXTM010000003	2505	4483	79.78	74.69	*clpC*	endopeptidase Clp ATP-binding chain C	Stress survival (VFC0282)
JBTXTM010000003	31,919	33,109	99.49	72.85	*tufA*	elongation factor Tu	Adherence (VFC0001)
JBTXTM010000005	8994	10,625	98.83	70.14	*groEL*	chaperonin GroEL	Adherence (VFC0001)
JBTXTM010000009	414,397	415,450	75.41	73.3	*capB*	CapB involved in Poly-gamma-glutamate synthesis	Immune modulation (VFC0258)
JBTXTM010000009	415,516	415,965	99.33	75.5	*capC*	CapC involved in Poly-gamma-glutamate synthesis	Immune modulation (VFC0258)
JBTXTM010000011	36,265	37,356	77.71	73.18	*capB*	CapB involved in Poly-gamma-glutamate synthesis	Immune modulation (VFC0258)
JBTXTM010000011	37,395	37,813	93.11	73.27	*capC*	CapC involved in Poly-gamma-glutamate synthesis	Immune modulation (VFC0258)
JBTXTM010000033	126,318	126,877	93.13	75.71	*clpP*	ATP-dependent Clp protease proteolytic subunit	Stress survival (VFC0282)

**Table 5:** Secondary metabolites gene clusters analysis predicted by AntiSMASH.

Contig	Region	Genomic Coordinates	Predicted Product	Cluster Completeness	Contig Boundary Truncation
**A1**					
NZ_JBTXTN010000001.1	Region 1	88,887–110,785 (+)	terpene	Complete	No
NZ_JBTXTN010000001.1	Region 2	159,219–200,316 (+)	T3PKS	Complete	No
NZ_JBTXTN010000001.1	Region 3	380,542–401,432 (+)	terpene-precursor	Complete	No
NZ_JBTXTN010000003.1	Region 1	68,268–134,732 (+)	terpene-precursor, NRP-metallophore, NRPS	Complete	No
NZ_JBTXTN010000004.1	Region 1	16,106–42,331 (+)	lanthipeptide-class-i	Complete	No
NZ_JBTXTN010000004.1	Region 2	160,875–181,633 (+)	CDPS	Complete	No
NZ_JBTXTN010000005.1	Region 1	134,803–197,570 (+)	sactipeptide, other	Complete	No
NZ_JBTXTN010000007.1	Region 1	181,060–202,758 (+)	epipeptide	Complete	No
NZ_JBTXTN010000011.1	Region 1	182,199–210,152 (+)	NRPS	Incomplete (Fragmented)	Yes
NZ_JBTXTN010000012.1	Region 1	1–10,862 (+)	NRPS	Incomplete (Fragmented)	Yes
NZ_JBTXTN010000013.1	Region 1	1–26,771 (+)	NRPS	Incomplete (Fragmented)	Yes
NZ_JBTXTN010000019.1	Region 1	42,965–63,771 (+)	terpene	Complete	No
NZ_JBTXTN010000020.1	Region 1	225,678–340,534 (+)	transAT-PKS, PKS-like, T3PKS, NRPS	Complete	No
NZ_JBTXTN010000020.1	Region 2	396,884–474,106 (+)	NRPS, betalactone, transAT-PKS	Complete	No
**F6**					
NZ_JBTXTM010000009.1	Region 1	20,609–41,457 (+)	terpene	Complete	No
NZ_JBTXTM010000009.1	Region 2	180,046–197,469 (+)	phosphonate	Complete	No
NZ_JBTXTM010000010.1	Region 1	19,571–39,837 (+)	RRE-containing	Complete	No
NZ_JBTXTM010000012.1	Region 1	263,997–305,082 (+)	T3PKS	Complete	No
NZ_JBTXTM010000012.1	Region 2	499,896–511,332 (+)	RiPP-like	Complete	No
NZ_JBTXTM010000019.1	Region 1	99,295–121,163 (+)	terpene	Complete	No
NZ_JBTXTM010000031.1	Region 1	52,495–73,313 (+)	terpene	Complete	No
NZ_JBTXTM010000031.1	Region 2	23,0676–265,251 (+)	NI-siderophore	Complete	No
NZ_JBTXTM010000032.1	Region 1	14,517–35,413 (+)	terpene-precursor	Complete	No

**Table 6:** Resistance to simulated gastric and/or intestinal conditions.

Strain		CFU/ml ^a^			
		Un-Treated	SGF ^b^	SIF ^b^	SGF + SIF ^b^
F6	cells	4.19 × 10^8^ ± 0.11	3.38 × 10^5^ ± 0.42 (3.10)	1.62 × 10^6^ ± 0.61 (2.42)	8.4 × 10^4^ ± 0.10 (3.69)
	spores	5.00 × 10^8^ ± 0.23	1.00 × 10^7^ ± 0.18 (1.69)	4.95 × 10^8^ ± 0.57 (0.004)	1.75 × 10^7^ ± 0.26 (1.46)
A1	cells	4.35 × 10^7^ ± 0.68	1.52 × 10^5^ ± 0.41 (2.46)	4.60 × 10^5^ ± 0.19 (1.97)	1.43 × 10^5^ ± 0.34 (2.49)
	spores	4.30 × 10^9^ ± 0.19	0.76 × 10^9^ ± 0.23 (0.75)	1.20 × 10^9^ ± 0.12 (0.55)	2.30 × 10^9^ ± 0.19 (0.27)

^a^ Data reported are the means of three independent experiments, and the reported variation is the SD; ^b^ the log reduction against un-treated values is reported in parentheses.

**Table 7:** Antimicrobial activity of cell-free supernatants from strains A1 and F6 ^a^.

Target Microorganism	A1-LB	F6-LB	A1-S7	F6-S7
*Enterococcus faecalis* ATCC 29212	+	-	-	-
*Streptococcus faecalis* ATCC 33186	-	-	-	-
*Stphylococcus aureus* ATCC 6538	+	-	-	-
*Priestia megaterium* QMB *1551*	+	-	-	-
*Listeria monocytogenes* ATCC 7644	+	-	+	-
*Escherichia coli* ATCC 25922	-	-	-	-
*Pseudomonas aeruginosa* ATCCv15691	-	-	-	-
*Salmonella enterica* subsp. *typhimurium* ATCC 14028	-	-	-	-
*Shigella sonnei* ATCC 25931	-	-	-	-
*Candida albicans* ATCC 14028	-	-	-	-
*Candida glabrata * ^b^	-	-	-	-
*Candida parapsilosis * ^b^	-	-	-	-
*Mycobacterium smegmatis* MC ^2^	-	-	-	-

^a^ “+” indicates an inhibition halo between 0.5 and 0.7 cm; “-” indicates no activity. ^b^ intestinal isolates gift of A. Tavanti from the University of Pisa.

## Data Availability

The whole-genome shotgun projects for *Priestia megaterium* strain F6 and *Bacillus spizizenii* strain A1 have been deposited at DDBJ/ENA/GenBank under BioProject accession number PRJNA1406710. The genome sequences are available under GenBank accession numbers JBTXTM000000000 and JBTXTN000000000, respectively, corresponding to BioSample accession numbers SAMN54800019 for strain F6 and SAMN54800018 for strain A1. The versions described in this paper are JBTXTM000000000.1 and JBTXTN000000000.1.

## Supplementary Materials

The following supporting information can be downloaded at: https://www.mdpi.com/article/10.3390/ijms27167364/s1.

## References

[1] Vittoria M., Saggese A., Isticato R., Baccigalupi L., Ricca E. (2023). Probiotics as an Alternative to Antibiotics: Genomic and Physiological Characterization of Aerobic Spore Formers from the Human Intestine. Microorganisms.

[2] Abouelela M.E., Helmy Y.A. (2024). Next-Generation Probiotics as Novel Therapeutics for Improving Human Health: Current Trends and Future Perspectives. Microorganisms.

[3] Lau L.Y.J., Quek S.Y. (2024). Probiotics: Health Benefits, Food Application, and Colonization in the Human Gastrointestinal Tract. Food Bioeng..

[4] Conrad N.L., Mazzoleni I., Abreu M.C., Costa A.V., Di Giácomo C.P., Zorzi V.S.G., Leite F.P.L. (2026). Horse Immune Response of Recombinant Lawsonia Intracellularis Vaccine: Assessing the Immunomodulatory Impact of Probiotic Supplementation. Res. Vet. Sci..

[5] Dong Y., Zhang Y., Xu F., Zou K. (2024). Extensive Genomic Characterization, Pre-Clinical Probiotic Evaluation, and Safety Analysis of *Bifidobacterium longum* Subsp. *longum* BL21 Isolated from Infant Feces. Microb. Pathog..

[6] Wang X., Wang W., Lv H., Zhang H., Liu Y., Zhang M., Wang Y., Tan Z. (2021). Probiotic Potential and Wide-Spectrum Antimicrobial Activity of Lactic Acid Bacteria Isolated from Infant Feces. Probiotics Antimicrob. Proteins.

[7] Soares M.B., Almada C.N., Pereira E.P.R., Ferreira B.M., Balthazar C.F., Khorshidian N., Rocha R.S., Xavier-Santos D., Cruz A.G., Ranadheera C.S. (2023). Review—Sporeforming Probiotic Bacteria: Characteristics, Health Benefits, and Technological Aspects for Their Applications in Foods and Beverages. Trends Food Sci. Technol..

[8] Fakhry S., Sorrentini I., Ricca E., De Felice M., Baccigalupi L. (2008). Characterization of Spore Forming Bacilli Isolated from the Human Gastrointestinal Tract. J. Appl. Microbiol..

[9] Hong H.A., Khaneja R., Tam N.M.K., Cazzato A., Tan S., Urdaci M., Brisson A., Gasbarrini A., Barnes I., Cutting S.M. (2009). *Bacillus subtilis* Isolated from the Human Gastrointestinal Tract. Res. Microbiol..

[10] Cutting S.M. (2011). *Bacillus* Probiotics. Food Microbiol..

[11] Colom J., Freitas D., Simon A., Brodkorb A., Buckley M., Deaton J., Winger A.M. (2021). Presence and Germination of the Probiotic *Bacillus subtilis* DE111^®^ in the Human Small Intestinal Tract: A Randomized, Crossover, Double-Blind, and Placebo-Controlled Study. Front. Microbiol..

[12] Mai J., Liang B., Xiong Z., Ai X., Gao F., Long Y., Yao S., Liu Y., Gong S., Zhou Z. (2019). Oral Administration of Recombinant *Bacillus subtilis* Spores Expressing *Helicobacter pylori* Neutrophil-activating Protein Suppresses Peanut Allergy via Up-regulation of Tregs. Clin. Exp. Allergy.

[13] Majeed M., Nagabhushanam K., Mundkur L., Paulose S., Divakar H., Rao S., Arumugam S. (2023). Probiotic Modulation of Gut Microbiota by *Bacillus coagulans* MTCC 5856 in Healthy Subjects: A Randomized, Double-Blind, Placebo-Control Study. Medicine.

[14] Jung H.S., Lee H.W., Kim K.T., Lee N.K., Paik H.D. (2024). Anti-Inflammatory, Antioxidant Effects, and Antimicrobial Effect of *Bacillus subtilis* P223. Food Sci. Biotechnol..

[15] Spagnuolo M.S., Petecca N., De Palma F., Troise A.D., Di Porzio A., Barrella V., Saggese A., De Pascale S., De Stefano M., Scaloni A. (2026). Gut-Brain Axis: Beneficial Impact of *Shouchella clausii* Spores on Fructose Induced Dysfunction Is Associated with Modulation of the Deoxycholic Acid–TGR5 Pathway. Mol. Med..

[16] Di Porzio A., Barrella V., Saggese A., Baccigalupi L., Cigliano L., Ricca E., Iossa S., Mazzoli A. (2025). Fructose-Induced Impairment of Liver and Skeletal Muscle Metabolism Is Prevented by Administration of *Shouchella clausii* Spores by Preserving Mitochondrial Function and Insulin Sensitivity. Mol. Nutr. Food Res..

[17] Vittoria M., Horwell E., Bastoni D., Saggese A., Baccigalupi L., Cutting S.M., Ricca E. (2024). *Bacillus subtilis* SF106 and *Bacillus clausii* SF174 Spores Reduce the Inflammation and Modulate the Gut Microbiota in a Colitis Model. Benef. Microbes.

[18] Dias R.S., Martinez D.P., Leite F.P.L., Avila L.F.D.C.D., Kremer F.S. (2025). Pato: Prediction of Probiotic Bacteria Using Metabolic Features. Braz. J. Microbiol..

[19] Galanis A., Papadimitriou K., Moloney G.M. (2025). Editorial: Omics Technologies and Bioinformatic Tools in Probiotic Research. Front. Microbiol..

[20] (2012). EFSA Panel on Additives and Products or Substances used in Animal Feed (FEEDAP). Guidance on the Assessment of Bacterial Susceptibility to Antimicrobials of Human and Veterinary Importance. EFSA J..

[21] Allende A., Alvarez-Ordóñez A., Bortolaia V., Bover-Cid S., De Cesare A., Dohmen W., Guillier L., Jacxsens L., Nauta M., EFSA Panel on Biological Hazards (BIOHAZ) (2026). Update of the List of QPS-recommended Biological Agents Intentionally Added to Food or Feeds as Notified to EFSA. EFSA J..

[22] Conrad R.E., Viver T., Gago J.F., Hatt J.K., Venter S.N., Rossello-Mora R., Konstantinidis K.T. (2022). Toward Quantifying the Adaptive Role of Bacterial Pangenomes during Environmental Perturbations. ISME J..

[23] Hyun J.C., Palsson B.O. (2023). Reconstruction of the Last Bacterial Common Ancestor from 183 Pangenomes Reveals a Versatile Ancient Core Genome. Genome Biol..

[24] Whelan F.J., Hall R.J., McInerney J.O. (2021). Evidence for Selection in the Abundant Accessory Gene Content of a Prokaryote Pangenome. Mol. Biol. Evol..

[25] Tonkin-Hill G., Gladstone R.A., Pöntinen A.K., Arredondo-Alonso S., Bentley S.D., Corander J. (2023). Robust Analysis of Prokaryotic Pangenome Gene Gain and Loss Rates with Panstripe. Genome Res..

[26] Wu Y.N., Wu S.R. (2025). Polyglutamic Acid as an Antiviral Agent: Mechanistic and Structural Insights. Pharmaceutics.

[27] Candela T., Mock M., Fouet A. (2005). CapE, a 47-Amino-Acid Peptide, Is Necessary for *Bacillus anthracis* Polyglutamate Capsule Synthesis. J. Bacteriol..

[28] Youngman P., Perkins J.B., Losick R. (1984). Construction of a Cloning Site near One End of Tn917 into Which Foreign DNA May Be Inserted without Affecting Transposition in *Bacillus subtilis* or Expression of the Transposon-Borne Erm Gene. Plasmid.

[29] Eppinger M.B., Bunk B., Johns M.A., Edirisinghe J.N., Kutumbaka K.K., Koenig S.S.K., Creasy H.H., Rosovitz M.J., Riley D.R., Daugherty S. (2011). Genome Sequences of the Biotechnologically Important *Bacillus megaterium* Strains QM B1551 and DSM319. J. Bacteriol..

[30] Saggese A., Giglio R., D’Anzi N., Baccigalupi L., Ricca E. (2022). Comparative Genomics and Physiological Characterization of Two Aerobic Spore Formers Isolated from Human Ileal Samples. Int. J. Mol. Sci..

[31] Li H., Han X., Zhang J., Dong Y., Xu S., Bao Y., Chen C., Feng Y., Cui Q., Li W. (2019). An Effective Strategy for Identification of Highly Unstable Bacillaenes. J. Nat. Prod..

[32] Sabino Y.N.V., de Araújo Domingues K.C., Mathur H., Gómez-Mascaraque L.G., Drouin G., Martínez-Abad A., Tótola M.R., Abreu L.M., Cotter P.D., Mantovani H.C. (2023). Exopolysaccharides Produced by *Bacillus* Spp. Inhibit Biofilm Formation by *Staphylococcus aureus* Strains Associated with Bovine Mastitis. Int. J. Biol. Macromol..

[33] Rychen G., Aquilina G., Azimonti G., Bampidis V., de Lourdes Bastos M.L., Bories G., Chesson A., Cocconcelli P.S., Flachowsky G., Gropp J. (2018). Guidance on the Characterisation of Microorganisms Used as Feed Additives or as Production Organisms. EFSA J..

[34] Gupta M., Rao K.K. (2009). Epr Plays a Key Role in DegU-Mediated Swarming Motility of *Bacillus subtilis*. FEMS Microbiol. Lett..

[35] Vittoria M., Saggese A., Barletta G.D.G., Castaldi S., Isticato R., Baccigalupi L., Ricca E. (2023). Sporulation Efficiency and Spore Quality in a Human Intestinal Isolate of *Bacillus Cereus*. Res. Microbiol..

[36] Cutting S., Vander Horn P.B., Harwood C., Cutting S. (1990). Genetic analysis. Molecular Biological Methods for Bacillus.

[37] Nicholson W.L., Setlow P., Harwood C., Cutting S. (1990). Sporulation, germination and outgrowth. Molecular Biological Methods for Bacillus.

[38] Saggese A., Isticato R., Cangiano G., Ricca E., Baccigalupi L. (2016). CotG-Like Modular Proteins Are Common among Spore-Forming *Bacilli*. J. Bacteriol..

[39] Lugli G.A., Fontana F., Tarracchini C., Milani C., Mancabelli L., Turroni F., Ventura M. (2023). MEGAnnotator2: A Pipeline for the Assembly and Annotation of Microbial Genomes. Microbiome Res. Rep..

[40] Piccoli C., Muñoz-Mérida A., Crottini A. (2024). PARSID: A Python Script for Automatic Analysis of Local BLAST Results for a Rapid Molecular Taxonomic Identification. BMC Res. Notes.

[41] Edgar R.C. (2004). MUSCLE: Multiple Sequence Alignment with High Accuracy and High Throughput. Nucleic Acids Res..

[42] Edgar R.C. (2022). Muscle5: High-Accuracy Alignment Ensembles Enable Unbiased Assessments of Sequence Homology and Phylogeny. Nat. Commun..

[43] Capella-Gutiérrez S., Silla-Martínez J.M., Gabaldón T. (2009). trimAl: A Tool for Automated Alignment Trimming in Large-Scale Phylogenetic Analyses. Bioinformatics.

[44] Steenwyk J.L., Buida T.J., Li Y., Shen X.-X., Rokas A. (2020). ClipKIT: A Multiple Sequence Alignment Trimming Software for Accurate Phylogenomic Inference. PLoS Biol..

[45] Page A.J., Cummins C.A., Hunt M., Wong V.K., Reuter S., Holden M.T.G., Fookes M., Falush D., Keane J.A., Parkhill J. (2015). Roary: Rapid Large-Scale Prokaryote Pan Genome Analysis. Bioinformatics.

[46] Sitto F., Battistuzzi F.U. (2020). Estimating Pangenomes with Roary. Mol. Biol. Evol..

[47] Lamkiewicz K., Barf L.-M., Sachse K., Hölzer M. (2024). RIBAP: A Comprehensive Bacterial Core Genome Annotation Pipeline for Pangenome Calculation beyond the Species Level. Genome Biol..

[48] Seemann T. Abricate. https://github.com/tseemann/abricate.

[49] Chen L., Yang J., Yu J., Yao Z., Sun L., Shen Y., Jin Q. (2005). VFDB: A Reference Database for Bacterial Virulence Factors. Nucleic Acids Res..

[50] Liu B., Zheng D., Zhou S., Chen L., Yang J. (2022). VFDB 2022: A General Classification Scheme for Bacterial Virulence Factors. Nucleic Acids Res..

[51] Blin K., Shaw S., Vader L., Szenei J., Reitz Z.L., Augustijn H.E., Cediel-Becerra J.D.D., de Crécy-Lagard V., Koetsier R.A., Williams S.E. (2025). antiSMASH 8.0: Extended Gene Cluster Detection Capabilities and Analyses of Chemistry, Enzymology, and Regulation. Nucleic Acids Res..

