# Supplementary material for: In Silico and In Vitro Characterization of Fecal Spore-Forming Bacteria with Probiotic Potential
Source: Int J Mol Sci. 2026 Aug 18;27(16):7364. doi: 10.3390/ijms27167364 (PMC13513138; doi:10.3390/ijms27167364)
Supplement: Supplementary file 1 [file ijms-27-07364-s001.zip › ijms-4441845-supplementary.pdf]

Supplementary Materials

Table S1. Strain list and hemolytic activity[a].

Individual sampled	Strain name	Hemolytic activity
1	F1	γ
1	F2	γ
1	F3	γ
1	F5	γ
1	F6	γ
1	F7	γ
1	F8	γ
1	F9	γ
1	F10	γ
1	F11	γ
2	A1	γ
2	A2	β
2	A3	β
2	A4	β
2	A5	β
2	A6	β
2	A7	β
3	Fa1a	γ
3	Fa1b	γ
3	Fa2	γ
3	Fa3	β
3	Fa4	β
4	Aa2a	β
4	Aa2c	β
4	Aa2d	β
4	Aa3a	β
4	Aa3c	β
5	Ma3a	β
5	Ma4a	β
5	Ma4b	β
5	Ma5a	β
5	Ma5b	β
5	Ma6a	β
5	Ma6b	β

[a] The hemolytic activity was performed in biological triplicate, using MV2 [Vittoria et al., 2023] as a positive control for β-hemolysis and *B. subtilis* MV24 [Vittoria et al., 2023] as a negative control for γ-hemolysis.

Table S2. Minimum inhibitory concentrations (MIC) [a, b] of spore-forming strains for eight selected antibiotics.

Strain [c]	Erythromycin	Vancomycin	Gentamicin	Clindamycin	Kanamycin	Streptomycin	Chloramphenicol	Tetracycline
F1	1.500	0.500	0.032	0.750	6.000	R	R	0.500
F2	0.125	0.250	2.500	0.750	1.000	R	R	1.000
F3	0.094	0.500	2.000	0.190	3.500	R	R	1.000
F5	0.064	0.750	0.750	0.380	1.500	R	R	0.500
F6	1.000	0.380	0.016	3.000	0.125	1.000	4.000	0.750
F7	0.094	0.094	0.190	0.380	0.380	R	R	R
F8	0.250	0.250	0.190	0.190	0.500	R	R	R
F9	0.190	0.190	0.500	0.025	0.250	R	R	R
F10	0.380	0.380	0.380	0.190	0.500	R	R	R
F11	1.500	1.500	0.250	0.190	0.380	R	R	R
A1	0.047	0.190	0.190	0.750	0.750	2.000	1.500	0.047
A2	0.064	0.190	0.125	0.190	0.380	R	2.000	3.000
A3	2.000	0.125	0.125	0.094	0.750	R	1.500	4.000
A4	1.500	R	0.750	0.380	6.000	7.000	4.000	0.500
A5	0.380	0.190	0.250	0.500	0.380	R	2.000	1.500
A6	0.250	0.190	0.500	0.380	0.500	R	2.000	2.000
A7	0.500	0.094	0.500	0.380	1.500	R	6.000	3.000
Fa1a	R	R	R	R	R	R	3.000	1.000
Fa1b	R	R	1.500	R	R	R	3.000	1.000
Fa2	R	R	R	R	R	R	4.000	R
Fa3	0.047	0.125	0.750	0.190	R	7.000	2.000	2.000
Fa4	0.094	0.250	1.000	0.190	1.500	R	1.500	1.500
Aa2a	0.190	0.094	0.500	0.380	1.000	R	0.750	1.000
Aa2c	0,064	0.190	0.190	0.380	0.750	R	1.000	0.500
Aa2d	0,250	0.380	0.750	0.190	1.500	R	1.500	1.500
Aa3a	R	R	2.000	R	6.000	R	2.500	2.000
Aa3c	0.250	0.380	1.000	0.125	1.000	R	2.000	1.500
Ma3a	0.064	3.000	0.500	0.125	0.750	R	0.750	1.500
Ma4a	0.074	0.125	1.000	4.000	1.000	R	1.000	1.000
Ma4b	0.125	1.000	0.750	0.190	2.000	R	0.500	2.000
Ma5a	0.125	0.750	0.750	0.500	3.000	R	1.500	4.000
Ma5b	0.094	0.190	0.190	0.190	0.750	R	0.750	0.750
Ma6a	0.047	0.750	0.500	1.000	0.750	R	3.000	1.500

Ma6b	0.125	0.500	0.190	0.250	0.750	R	0.750	4.000
**EFSAcut-off values**	**4.000**	**4.000**	**4.000**	**4.000**	**8.000**	**8.000**	**8.000**	**8.000**

[a] Values are µg/ml and are the mean of three independent experiments, range 0,016 – 256a µg/ml; [b] R indicates values equal or higher than the breakpoint; [c] Non-hemolytic strains are indicated in red. *Bacillus subtilis* MV24 strain was used as a negative control, while strains MV14, MV15, and MV17 served as positive controls [Vittoria et al., 2023].

Table S3. Accession number of the genome of the different bacterial strains used in the pangenomic analysis

Species	No. of Genomes	Accession Numbers List
***B. spizizenii* A1**	72	NZ_VOOM01000010.1;NZ_JBHUSU010000001.1;NZ_AJHN01000001.1;NZ_VBUQ01000001.1;NZ_CP097500.1;NZ_LSMU01000001.1;NC_016047.1;NZ_AJHM01000001.1;NZ_JARLZH010000001.1;NZ_CM126354.1;NZ_JARSKD010000001.1;NZ_MWZB01000100.1;NZ_JBCMPW010000001.1;NZ_JARSRA010000001.1;NZ_ADGS01000037.1;NZ_JARSSA010000001.1;NZ_VKQE01000095.1;NC_014479.1;NZ_JBLZQQ010000001.1;NZ_CP187554.1;NZ_JARTBF010000001.1;NZ_JBHFXE010000001.1;NZ_JAWVOG010000001.1;NZ_JARSKF010000001.1;NZ_JAMAWV010000100.1;NZ_JBDLZK010000001.1;NZ_CBCSHL010000001.1;NZ_JARSQS010000001.1;NZ_AFSG01000020.1;NZ_JARSMN010000001.1;NZ_JARSKI010000001.1;NZ_AJHL01000001.1;NZ_JAHHED010000035.1;NZ_CBCRWV010000001.1;NZ_JAKYKB010000994.1;NZ_JARSMO010000001.1;NZ_CP148102.1;NZ_JBLZQP010000001.1;NZ_JARSRI010000001.1;NZ_CP145137.1;NZ_JAVIYK010000001.1;NZ_JARSQC010000001.1;NZ_JBCMSL010000001.1;NZ_JANLFG010000001.1;NZ_CP034943.1;NZ_JACJGE010000095.1;NZ_NKAK01000010.1;NZ_CP176536.1;NZ_UAQD01000014.1;NZ_JARSPU010000001.1;NZ_KN049967.1;NZ_MUXL01000001.1;NZ_JBCMZH010000001.1;NZ_CP167793.1;NZ_JARSKE010000001.1;NZ_JARSMM010000001.1;NZ_JARSOH010000001.1;NZ_CP011051.1;NZ_CP118021.1;NZ_BJUC01000001.1;NZ_JARSKV010000001.1;NZ_JARSKC010000001.1;NZ_JARSQD010000001.1;NZ_JARSKH010000001.1;NZ_JARSKG010000001.1;NZ_JARSKW010000001.1;NZ_JARLXB010000001.1;NZ_AJHO01000001.1;NZ_JBCMQM010000001.1;NZ_CP145722.1;NZ_JARSMP010000001.1.
***P. megaterium* F6**	479	NZ_CP069397.1;NZ_JBPPNL010000001.1;NZ_CP189798.1;NZ_NUQA01000091.1;NZ_NTVG01000100.1;NZ_CP058262.1;NZ_NUFU01000011.1;NZ_JARHXG010000010.1; NZ_JANIOE010000010.1;NZ_JBALPV010000001.1;NZ_JBALPY010000001.1;NZ_JAXCWO010000001.1;NZ_JAHWCQ010000001.1;NZ_VKPR01000100.1;NZ_JBPCHL010000001.1;NZ_NULX01000010.1;NZ_JARMWB010000001.1;NZ_CP041066.1;NZ_CP085431.1;NZ_JBALNW010000001.1;NZ_JARMWH010000001.1;NZ_JBANBS010000001.1;NZ_JBQCMI010000001.1;NZ_JBALMN010000001.1;NZ_JBJYUA010000001.1;NZ_JBNPWY010000001.1;NZ_KN125581.1;NZ_JARMWP010000001.1;NZ_JARSFE010000001.1; NZ_VXLA01000106.1;NZ_NTZT01000010.1;NZ_JBSHHG010000001.1;NZ_JAPZUM010000001.1;NZ_CP127874.1;NZ_JABAKC010000018.1;NZ_CM129583.1;NZ_CAXOOG010000001.1;NZ_JAWPHI010000011.1;NZ_CP187630.1;NZ_CP162497.1;NZ_JAOEFZ010000034.1;NZ_NTWH01000010.1;NZ_JALAUQ010000001.1;NZ_JBALLQ010000001.1;NZ_JAIQVI010000001.1;NZ_JBLURS010000010.1;NZ_JBDLZE010000001.1;NZ_JBNMRW010000001.1;NZ_CM125657.1;NZ_CM125650.1;NZ_CDRUCM010000001.1;NZ_CP130477.1;NZ_JBQCMA010000001.1;NZ_NVCR01000010.1;NZ_JBNNNE010000001.1;NZ_JANBML010000001.1;NZ_JAHTKR010000001.1;NZ_JARMTD010000001.1;NZ_JARMTO010000001.1;NZ_CP189780.1;NZ_JBALLP010000001.1;NZ_JBEUTV010000001.1;NZ_CP141294.1;NZ_JBALQD010000001.1;NZ_JAUKEO010000001.1;NZ_JBAWKX010000001.1;NZ_JBEAFA010000001.1;NZ_NUHQ01000010.1;NZ_CM125967.1;NZ_JAUKEM010000001.1;NZ_JBALOM010000001.1;NZ_JBPCHM010000001.1;NZ_NUWI01000007.1;NZ_CP058255.1;NZ_JBALLR010000001.1;NZ_JBANBV010000001.1;NZ_JARMWD010000001.1;NZ_JBALQT010000001.1;NZ_JAQQBQ010000004.1;NZ_JAUIQX010000001.1;NZ_JALDXD010000001.1;NZ_JARMTA010000001.1;NZ_CP146257.1;NZ_JBANCD010000001.1;NZ_JBALLU010000001.1;NZ_JAJOOU010000001.1;NZ_MRUF01000001.1;NZ_JBGLYW010000099.1;NZ_CP022674.1;NZ_JBALQP010000001.1;NZ_OZ014518.1;NZ_JARRUG010000001.1;NZ_JAUUTO010000001.1;NZ_JARVCR010000001.1;NZ_JAOTOY010000001.1;NZ_CP150963.1;NZ_JAIFON010000001.1;NZ_NUXL01000010.1;NZ_JAUKEL010000001.1;NZ_UFSR01000004.1;NZ_JARMUU010000001.1;NZ_RYZX01000100.1;NZ_JARMTE010000001.1;NZ_JBDOIM010000009.1;NZ_JBHVVH010000001.1;NZ_JBRJIC010000001.1;NZ_NVLN01000010.1;NZ_CDRMVY010000001.1;NZ_JBHWVF010000001.1;NZ_NVBQ01000009.1;NZ_CP169655.1;NZ_CP180490.1;NZ_JBFRBG010000001.1;NZ_JARMUV010000001.1;NZ_NUIN01000010.1;NZ_NUZS01000008.1;NZ_JBQCKG010000001.1;NZ_JBEUTR010000001.1;NZ_JACYNX010000001.1;NZ_JAIWYW010000010.1;NZ_JBALOU010000001.1;NZ_JAQZSE010000001

	.1;NZ_JBALNE010000001.1;NZ_JAFEMS010000001.1;NZ_CP025700.1;NZ_JALJWM010000001.1;NZ_JBALPS010000001.1;NZ_UFSS01000007.1;NZ_NGUL01000001.1;NZ_JBHVDJ010000001.1;NZ_JBALNM010000001.1;NZ_JARMTK010000001.1;NZ_JAKKUX010000010.1;NZ_JARMWO010000001.1;NZ_JACYVS010000001.1;NZ_JARTIP010000001.1;NZ_JBEUTQ010000001.1;NZ_CAKKMH010000001.1;NZ_JBALLX010000001.1;NZ_WIPB01000100.1;NZ_JAUCND010000001.1;NZ_NKAQ01000001.1;NZ_JBKIKC010000001.1;NZ_CP010586.1;NZ_JARMUT010000001.1;NZ_NTUU01000075.1;NZ_JBQLIP010000001.1;NZ_JARMVL010000001.1;NZ_JAUUTT010000001.1;NZ_JBDLZN010000001.1;NC_014103.1;NZ_JBALNU010000001.1;NZ_JAMATO010000099.1;NZ_CP187617.1;NZ_JBKQXB010000001.1;NZ_JARVWM010000002.1;NZ_VKPT01000095.1;NZ_JAVDWZ010000001.1;NZ_JBIQEA010000010.1;NZ_JARMTZ010000001.1;NZ_JARTIO010000001.1;NZ_JBALPO010000001.1;NZ_CP032527.2;NZ_NUKD01000098.1;NZ_JARTIY010000001.1;NZ_JAOTPA010000003.1;NZ_NUIA01000009.1;NZ_JBBLUG010000001.1;NZ_CP129891.1;NZ_JARMUS010000001.1;NZ_CP009920.1;NZ_NUON01000097.1;NZ_JAUKEK010000001.1;NZ_JAUELX010000004.1;NZ_JBJNVQ010000008.1;NZ_QDFM01000001.1;NZ_JBANBO010000001.1;NZ_JALAUS010000001.1;NZ_JBALQB010000001.1;NZ_JAJTTM010000010.1;NZ_JBHVVG010000001.1;NZ_JARMUI010000001.1;NZ_JANIPL010000010.1;NZ_CM125445.1;NZ_JARMTF010000001.1;NZ_CM129582.1;NZ_JACAFM010000001.1;NZ_CP187646.1;NZ_JAUUTS010000001.1;NZ_CP031777.1;NZ_JAMAVH010000099.1;NZ_CM125648.1;NZ_VDTN01000010.1;NZ_JBBJSO010000001.1;NZ_REFM01000005.1;NZ_JARVWN010000001.1;NZ_CP065335.1;NZ_CP174470.1;NZ_JARMUB010000001.1;NZ_CM132624.1;NZ_JBHJZR010000001.1;NZ_JAUSYE010000001.1;NZ_JBALQA010000001.1;NZ_JBANCB010000001.1;NZ_VDZC01001000.1;NZ_JBBPHV010000001.1;NZ_SSWM01000001.1;NZ_JARHXD010000010.1;NZ_JBALQH010000001.1;NZ_JBEVAE010000078.1;NZ_CP047699.1;NZ_JAMAUT010000100.1;NZ_JABXFF010000020.1;NZ_CP048883.1;NZ_JBSHGJ010000001.1;NZ_QDFP01000001.1;NZ_JAHHZD010000010.1;NZ_NUPF01000008.1;NZ_JBALPW010000001.1;NZ_JBBCLA010000001.1;NZ_JAYIXA010000001.1;NZ_NUMB01000008.1;NZ_CP169245.1;NZ_JARHWZ010000010.1;NZ_JASVWC010000001.1;NZ_JARMWM010000001.1;NZ_SUPM01000010.1;NZ_NVEG01000010.1;NZ_JBALMJ010000001.1;NZ_CP120618.2;NZ_LUCO01000079.1;NZ_JBALLW010000001.1;NZ_PNHN01000001.1;NZ_JARMTN010000001.1;NZ_JBHYKS010000001.1;NZ_QDFU01000001.1;NZ_CM125647.1;NZ_JAHVMP010000100.1;NZ_JAWPPN010000001.1;NZ_JAQQBJ010000001.1;NZ_JBALNV010000001.1;NZ_CP023317.1;NC_017138.1;NZ_NUZX01000005.1;NZ_JASPFG010000001.1;NZ_JBNNJY010000001.1;NZ_JBALOB010000001.1;NZ_JARMVA010000001.1;NZ_NTVK01000010.1;NZ_CATKQG010000001.1;NZ_NUKI01000056.1;NZ_JARMWL010000001.1;NZ_NUWY01000010.1;NZ_JBALLF010000001.1;NZ_CP035094.1;NZ_CP170487.1;NZ_JBALOE010000001.1;NZ_NVMV01000010.1;NZ_POTF01000001.1;NZ_VDST01000100.1;NZ_JBDIZI010000001.1;NZ_QDFO01000001.1;NZ_JBEUTS010000001.1;NZ_NUZT01000010.1;NZ_JBEUMJ010000010.1;NZ_JAHAVB010000010.1;NZ_CAVNYK010000001.1;NZ_JARMUA010000001.1;NZ_JBALPL010000001.1;NZ_BSYK01000001.1;NZ_JARMWN010000001.1;NZ_VEEN01000100.1;NZ_NTTN01000010.1;NZ_NVDJ01000007.1;NZ_JAMAYJ010000010.1;NZ_JBALNK010000001.1;NZ_JBHVSP010000001.1;NZ_LFQJ01000001.1;NZ_VECD01000099.1;NZ_NUKX01000005.1;NZ_NTTT01000031.1;NZ_NVNE01000100.1;NZ_JBBCKQ010000001.1;NZ_VYTX01000001.1;NZ_CM125446.1;NZ_JBALNO010000001.1;NZ_CP071466.1;NZ_CP109761.1;NZ_CP026736.1;NZ_JBMWAL010000001.1;NZ_CM003163.1;NZ_JBSEDV010000001.1;NZ_JBALQC010000001.1;NZ_CP187666.1;NC_014019.1;NZ_CP064270.1;NZ_NUVK01000010.1;NZ_WWFB01000001.1;NZ_JAUKEP010000001.1;NZ_CP120608.2;NZ_CP028084.1;NZ_NUWZ01000008.1;NZ_NUVY01000024.1;NZ_JBALOJ010000001.1;NZ_JBFEGB010000010.1;NZ_NUKK01000006.1;NZ_CDRMVV010000001.1;NZ_JBANBZ010000001.1;NZ_JAVDWX010000001.1;NZ_LDPJ01000001.1;NZ_JBHEYT010000001.1;NZ_NVKN01000065.1;NZ_JBSHHH010000001.1;NZ_JBALLZ010000001.1;NZ_JAYKZT010000001.1;NZ_LODM01000001.1;NZ_JBEUUC010000001.1;NZ_JACZID010000100.1;NZ_JAQQBP010000001.1;NZ_NVKU01000061.1;NZ_JARMWW010000001.1;NZ_CP151780.1;NZ_CP098610.1;NZ_JARMWG010000001.1;NZ_NTRV01000088.1;NZ_NUIR01000085.1;NZ_JBBJSQ010000001.1;NZ_JARMWV010000001.1;NZ_JBEUTU010000001.1;NZ_JARMTV010000001.1;NZ_JARMUQ010000001.1;NZ_JBEUMI010000010.1;NZ_CP107543.1;NZ_VEEA01000010.1;NZ_NUWU01000043.1;NZ_JBALMM010000001.1;NZ_JBDIZP010000001.1;NZ_JARMWI010000001.1;NZ_CP117689.1;NZ_CP018874.1;NZ_NTYW01000099.1;NZ_JARMWF010000001.1;NZ_JAIQVO010000001.1;NZ_JACLPW010000001.1;NZ_JARMWK010000001.1;NZ_NUKH01000072.1;NZ_JAUOOU010000001.1;NZ_JBNQEJ010000001.1;NZ_JBHYLL010000001.1;NZ_JAUUTN010000001.1;NZ_NGUM01000001.1;NZ_CP085426.1;NZ_CP180722.1;NZ_JARMUH010000001.1;NZ_JBALPN010000001.1;NZ_CP073301.1;NZ_JBFRBF010000001.1;NZ_CP059457.1;NZ_JBALLL010000001.1;NZ_JARMTM010000001.1;NZ_NTRQ01000010.1;NZ_JBEWQB010000001.1;NZ_CP015226.1;NZ_CP045272.1;NZ_JARAOX010000038.1;NZ_NUWQ01000042.1;NZ_VEAZ01000010.1;NZ_CP069606.1;NZ_CP064793.1;NZ_JBALPI010000001.1;NZ_JAVIKB010000010.1;NZ_JACYYN010000010.1;NZ_JBANBQ010000001.1;NZ_NVEP01000010.1;NZ_NUZZ01000100.1;NZ_JBNMSD010000001.1;NZ_CP157504.1;NZ_JBALOH010000001.1;NZ_JBALMX010000001.1;NZ_JBBCLB010000001.1;NZ_JAMAVR010000100.1;NZ_AZUJ01000001.1;NZ_JARMVF010000001.1;NZ_NUVO01000100.1;NZ_JAMAXO010000100.1;NZ_CANLZB010000001.1;NZ_JBHOHO010000056.1;NZ_JBMIHZ010000001.1;NZ_JARMWE010000001.1;NZ_JARMTJ010000001.1;NZ_JBNNSG010000001.1;NZ_NUZC01000008.1;NZ_VEAU01000100.1;NZ_JARMVB010000001.1;NZ_JBEPMZ010000001.1;NZ_JARTIN010000001.1;NZ_JARMWC010000001.1;NZ_PRKV01000001.1;NZ_CATKPS010000001.1;NZ_LRPG01000001.1;NZ_JAHHXE010000010.1;NZ_CP064275.1;NZ_CP187621.1;NZ_JAZDWX010000010.1;NZ_JBANBR010000001.1;NZ

	_JAUERA010000001.1;NZ_JARRUE010000001.1;NZ_CP049296.1;NZ_JBALOD010000001.1;NZ_CP150954.1;NZ_JBALPF010000001.1;NZ_CP065213.1;NZ_JAOTPB010000001.1;NZ_CP069288.1;NZ_JARMWA010000001.1;NZ_JBPZPF010000001.1;NZ_CP109859.1;NZ_NTWY01000007.1;NZ_NUOI01000004.1;NZ_JBALQJ010000001.1;NZ_JBNCAS010000011.1;NZ_JJMH01000001.1;NZ_NTZG01000006.1;NZ_JAHHYI010000010.1;NZ_JBALOC010000001.1;NZ_JAMAUB010000100.1;NZ_BCVB01000001.1;NZ_JABBNK010000001.1;NZ_CP092387.1;NZ_JBANBY010000001.1;NZ_NVKD01000065.1;NZ_CP085415.1;NZ_JAGGNN010000009.1;NZ_JBIQEB010000010.1;NZ_JBSGRF010000001.1;NZ_LDEZ01000001.1;NZ_JARMWT010000001.1;NZ_JARMWJ010000001.1;NZ_CP140267.1;NZ_CP031776.1;NZ_JARAOW010000001.1;NZ_JBALMI010000001.1;NZ_JBALOF010000001.1;NZ_JAZDWE010000001.1;NZ_CM125649.1;NZ_NTYL01000010.1;NZ_CP120609.2;NZ_JASBCR010000001.1;NZ_NQLQ01000001.1;NZ_CM125656.1;NZ_JBITVQ010000001.1;NZ_JARHWY010000010.1;NZ_JBREIO010000001.1;NZ_JAMAVD010000099.1;NZ_JBHVTO010000001.1;NZ_JBALPM010000001.1;NZ_CP137800.1;NZ_CP130958.1;NZ_CP085442.1;NZ_JBKQAS010000001.1;NZ_JBEULA010000001.1;NZ_NVAW01000017.1;NZ_JBHXUP010000001.1;NZ_JAWDGZ010000001.1;NZ_JAUKEJ010000001.1;NZ_JAOTOZ010000001.1;NZ_JBALRN010000001.1;NZ_NUOQ01000066.1;NZ_JBHYKR010000001.1;NZ_CM125449.1;NZ_JBLOIX010000010.1;NZ_NUFL01000004.1;NZ_JBALPH010000001.1;NZ_NUKU01000010.1;NZ_PQWM01000001.1;NZ_CP123578.1;NZ_JBHVHM010000001.1;NZ_JARMVD010000001.1;

Table S4. Antimicrobial resistance genes identified by CARD analysis in the genomes of strains A1 and F6.

#FILE	SEQUENCE	START	END	GENE	% COVERAGE	% IDENTITY	ACCESSION	PRODUCT	RESISTANCE[a]
A1	JBTXTN010000001	359316	360481	*bmr*	99,66	91,85	M33768.1:194-1364	MFS antibiotic efflux pump.	disinfecting_agents_and_antiseptics; fluoroquinolone; nucleoside;
A1	JBTXTN010000001	535239	536441	*blt*	100	94,85	L32599.1:1236-2439	MFS efflux pump	disinfecting_agents_and_antiseptics; fluoroquinolone
A1	JBTXTN010000011	98352	99272	*mphK*	100	87,08	CP010314.1:275837-276758	Macrolide phosphotransferase	macrolide
A1	JBTXTN010000011	112282	113715	*lmrB*	100	92,68	JYFL01000006.1:113503-112069	Efflux pump	lincosamide; nucleoside
A1	JBTXTN010000011	161807	162400	*tmrB*	100	89,06	AL009126.3:339749-339155	ATP-binding tunicamycin resistance protein	nucleoside
A1	JBTXTN010000014	16420	18044	*vmlR*	98,84	92	NC_000964.3:606379-604735	ABC-F ATPase ribosomal protection protein	antibiotic_without_defined_classification; lincosamide; nucleoside; pleuromutilin;s treptogramin; streptogramin_A; streptogramin_B.
A1	JBTXTN010000016	195000	197570	*mprF*	100	91,6	AL009126.3:916777-919348	Integral membrane protein.	peptide
A1	JBTXTN010000019	262161	262499	*ykkC*	100	90,27	AL009126.1:1376516-1376855	Subunit of the YkkCD efflux pump.	aminoglycoside; tetracycline

A1	JBTXTN010000019	262499	262816	*ykkD*	100	94,34	AL009126.1:1376854-1377172	Subunit of the YkkCD efflux pump.	aminoglycoside; tetracycline
A1	JBTXTN010000020	375305	375725	*fosM1*	99,53	72,92	BK012111.1:0-423	Thiol transferase	phosphonic_acid
F6	JBTXTM010000017	8972	11555	*rphB*	96,99	75,44	KX531052.1:0-2655	Rifampin phosphotransferase	rifamycin
F6	JBTXTM010000021	41909	42435	*vatH*	80,03	74,53	GQ205627.2:3036-3687	Acetyltransferase	streptogramin; streptogramin_A

[a] Potential resistance to the indicate antimicrobials.

Reference

Vittoria, M.; Saggese, A.; Isticato R.; Baccigalupi, L.; Ricca, E. Probiotics as an Alternative to Antibiotics: Genomic and Physiological Characterization of Aerobic Spore Formers from the Human Intestine. Microorganisms. 2023 Jul 31;11(8):1978. doi: 10.3390/microorganisms11081978.
